# Strategies and mechanisms of precision genome engineering: From gene editing to genome writing

**DOI:** 10.1002/imo2.70115

**Published:** 2026-07-26

**Authors:** Kerui Huang, Jianhong Tian, Wenyan Zhao, Siqin Zhang, Lixuan Xiang, Meiqing Yang, Lu Liu, Yazhou Huang, Ningyun Zhang, Lei Sun, Peng Xie, Nanyu Kuang, Hong Zhou, Chengfeng Qiu, Jun Yan, Xiaocheng Jiang, Qiang Tu, Yun Wang, Haoliang Hu, Youming Zhang

**Affiliations:** ^1^ State Key Laboratory of Microbial Technology Shandong University Qingdao China; ^2^ State Key Laboratory of Microbial Technology (Changde R&D Base), College of Synthetic Biology Industry, Institute of Synthetic Biology Industry Hunan University of Arts and Science Changde China; ^3^ College of Life Sciences Hunan Normal University Changsha China; ^4^ School of Life Sciences Sun Yat‐Sen University Guangzhou China; ^5^ Central South University Xiangya School of Medicine Affiliated Changde Hospital (The first people's hospital of Changde city) Changde China; ^6^ Shanghai Collaborative Innovation Center of Plant Germplasm Resources Development, Shanghai Engineering Research Center of Plant Germplasm Resources College of Life Sciences, Shanghai Normal University Shanghai China; ^7^ Chinese Medicinal Materials Breeding Innovation Center of Yuelushan Laboratory Changsha China; ^8^ Institute of Science and Technology for Brain‐Inspired Intelligence, Fudan University Shanghai China; ^9^ Neuroimaging Research Branch, National Institute on Drug Abuse National Institutes of Health Baltimore Maryland USA; ^10^ Department of Radiology, The First Affiliated Hospital, Hengyang Medical School University of South China Hengyang China; ^11^ Evidence‐Based Medicine and Clinical Research Center Hunan University of Medicine General Hospital Huaihua China; ^12^ Hunan Provincial Demonstration Center of Forestry Seedling Breeding Changsha China; ^13^ Yuelushan Laboratory Changsha China; ^14^ The Key Laboratory of Study and Discovery of Small Targeted Molecules of Hunan Province, School of Pharmaceutic Science, Health Science Center Hunan Normal University Changsha China

**Keywords:** CRISPR systems, DNA repair, genome editing, genome writing, synthetic biology

## Abstract

Genomic manipulation has advanced from stochastic nuclease‐mediated disruption toward programmable, deterministic precision. Early clustered regularly interspaced short palindromic repeats (CRISPR) strategies enabled targeted mutagenesis through double‐strand breaks; however, their therapeutic application is limited by genotoxicity, chromosomal instability, and dependence on endogenous repair pathways that are difficult to predict. In this review, we examined the transition from gene editing to genome writing, an approach that decouples genomic modification from host repair pathways to better balance efficiency, precision, and payload delivery. We also discussed the principles of precision technologies, including base and prime editors, and described emerging large‐scale writers, such as CRISPR‐associated transposases and recombinase‐based bridge RNAs, which enable the integration of multi‐kilobase synthetic modules. Beyond enzymatic mechanisms, we further considered the combined use of generative artificial intelligence, structural biology, and novel delivery architectures as potential strategies to overcome current biological limitations. Taken together, these developments point toward Generative Biology, in which computational design and high‐throughput screening transform the genome from a static substrate into a more dynamic model for complex, synthetic functional design.

## Highlights

- Genome manipulation is moving from nuclease‐mediated, stochastic disruption toward deterministic, programmable genome writing.
- Precision technologies, such as base and prime editors, minimize genotoxicity by bypassing endogenous double‐strand break repair.
- RNA‐guided platforms, including CRISPR‐associated transposases and bridge RNAs, enable seamless insertion of multi‐kilobase modules, thereby overcoming cargo limitations.
- Generative Artificial Intelligence (AI), structural biology, and novel delivery architectures are revolutionizing the design of *de novo* synthetic effectors.
- These synergistic advancements are ushering in Generative Biology, transforming genomes into dynamically programmable media for synthetic design.

## Graphical abstract

In this review, we examined the progression of genome manipulation from stochastic nuclease‐mediated cutting toward precise editing and programmable genome writing. We discussed tools like multi‐kilobase RNA‐guided integrators and Artificial Intelligence (AI)‐designed effectors and showed how these advances enable researchers to treat genomes as programmable media for synthetic design and Generative Biology.

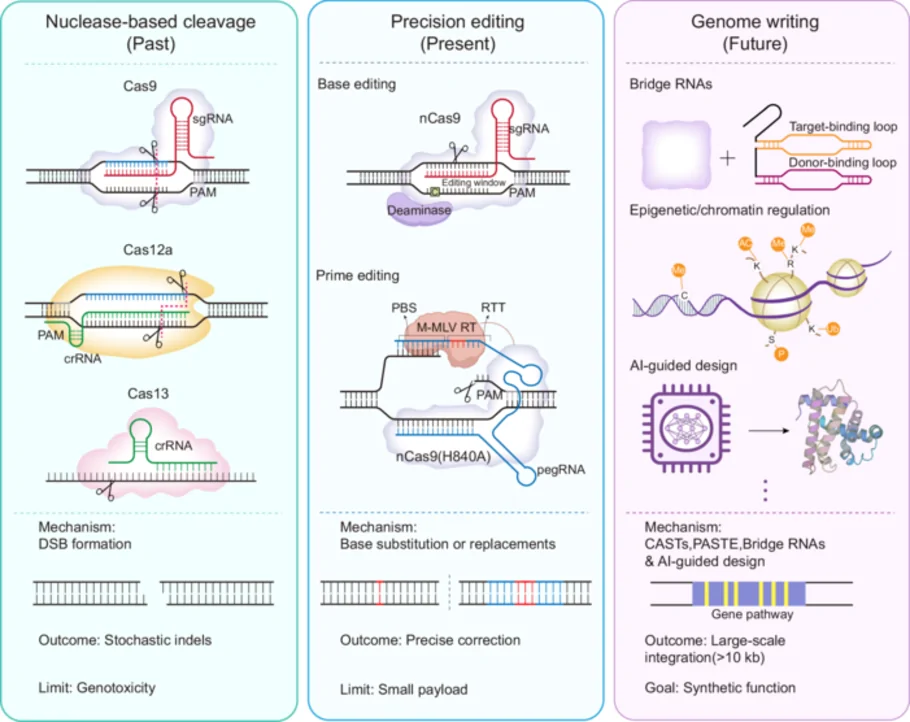

## 1 INTRODUCTION

The approaches to genomic manipulation have evolved from stochastic mutagenesis to programmable and precise methods. Early strategies based on zinc finger nucleases (ZFNs) and transcription activator‐like effector nucleases (TALENs) showed that targeted DNA modification was possible. However, the complex requirements of protein engineering limited their application in practice [1, 2, 3, 4]. Later, the CRISPR‐Cas9 system transformed this field. This system uses a single‐guide RNA (sgRNA) to direct the activity of endonucleases to specific DNA sequences [5, 6, 7]. This innate immune mechanism was adapted for mammalian genome editing [8, 9], and it established a scalable platform to perform targeted mutagenesis [9, 10, 11, 12]. These early strategies mainly depended on the induction of double‐strand breaks (DSBs) to trigger internal repair pathways. For example, they used nonhomologous end joining (NHEJ) for gene disruption and homology‐directed repair (HDR) for the insertion of sequences [13, 14, 15]. Although these methods are effective for gene ablation, the reliance on DSB‐mediated repair limits the potential for precision engineering. The NHEJ pathway has a stochastic nature, which leads to unpredictable insertion–deletion (indel) profiles. At the same time, the efficiency of HDR is quite limited in nondividing somatic tissues [16, 17, 18, 19]. In addition, the induction of DSBs can lead to substantial genotoxicity, such as p53‐mediated DNA damage responses [20, 21, 22] and large‐scale chromosomal deletions. They can also cause complex genomic rearrangements, which damage the genomic integrity [23, 24, 25, 26]. Thus, researchers urgently need new technologies to separate genomic modification from double‐strand cleavage.

With the development of related technologies, genomic manipulation has experienced a shift from genome editing to genome writing. Traditional genome editing mainly operates as a top‐down approach, and it relies on the localized alteration of existing genomic templates. Early systems heavily relied on nucleases to induce targeted DSBs and use stochastic endogenous repair, which restricts absolute control over the sequence [27, 28, 29, 30]. To solve this problem, advanced precision technologies have been developed to mediate direct chemical and enzymatic modifications. The development of cytosine and adenine base editors (CBEs/ABEs) enabled programmable nucleotide transitions without the generation of DSBs. This change minimizes the formation of indels and refines the editing paradigm [19, 31, 32, 33, 34].

On the other hand, genome writing operates as a different bottom‐up methodology. It gradually separates genetic manipulation from the constraints of natural templates, because it can deterministically install new genetic architectures. Prime editing initiated this transition. This method uses a template‐directed synthesis mechanism, which is mediated by a Cas9 nickase–reverse transcriptase fusion. It plays a role in writing specific insertions, deletions, and substitutions [35, 36]. To further expand the payload capacity for macroscopic writing, researchers developed several new technologies. For example, CRISPR‐associated transposases (CASTs), programmable addition via site‐specific targeting elements (PASTE) [37, 38, 39], and recombinase‐based bridge RNA (bRNAs) systems [40, 41] can help the programmable integration of multi‐kilobase genetic modules [29, 42, 43, 44, 45]. At the same time, the scope of precision engineering has expanded. It now includes the reversible modulation of transcriptional and epigenetic states [46, 47, 48]. In addition, scientists also use generative artificial intelligence (AI) to perform the de novo design of synthetic editing enzymes [49, 50, 51].

In this review, we critically examine the strategies and mechanisms underlying the transition from simple editing to complex genome writing. We discuss diverse molecular complexes from biophysical and structural biology perspectives. The primary aim of this review is to evaluate the strategic trade‐offs required to balance efficiency, precision, and payload capacity while overcoming biological barriers. Finally, we explore the emerging field of synthetic design, which uses generative AI and high‐throughput screening to create *de novo* editors and rewrite regulatory logic [49, 50, 52, 53, 54], thereby enabling biological functions to be written using computer code and advancing toward the synthesis and programmable engineering of entire genomes [55, 56, 57].

## 2 EVOLUTIONARY TRAJECTORY OF GENOMIC MANIPULATION

### Historical milestones in precision engineering

Genomic manipulation began with stochastic, labor‐intensive methods and has now progressed to programmable, deterministic precision. In other words, gene editing, the top‐down, small‐scale modification of existing sequences using nucleases, which inherently lacks locus‐scale sequence control [28, 45], has undergone a fundamental shift to genome writing, the bottom‐up, programmable insertion or *de novo* generation of new genetic content independent of host repair templates [27, 29]. Although studies using ZFNs and TALENs achieved targeted modifications, their applications were limited by complex protein‐engineering requirements [3, 4, 58]. However, the development of multiplex automated genome engineering (MAGE) in 2009 introduced accelerated evolution through simultaneous, large‐scale modifications, substantially advancing the field and foreshadowing the high‐throughput capabilities of modern systems [59, 60].

In 2012, the field of genome engineering advanced significantly when Jinek et al. characterized the biochemistry of the CRISPR‐Cas9 system from *Streptococcus pyogenes*. They showed that a synthetic sgRNA could program the Cas9 endonuclease to cleave DNA at specific sites. In this process, loading the RNA triggers a conformational change in the protein, which then activates its nuclease domains [6, 7, 61]. This work led to the CRISPR revolution in 2013, when several groups simultaneously reported successful genome editing in mammalian cells [8, 9, 12, 62, 63]. These early studies demonstrated that Cas9 could induce DSBs with efficiencies comparable to older tools while being substantially easier to design. Consequently, its application rapidly extended to plants and bacteria [6, 64, 65, 66, 67]. However, reliance on the endogenous NHEJ pathway made precise correction difficult to achieve. Although NHEJ is effective for gene disruption, such as in current efforts to reactivate fetal hemoglobin for sickle cell disease [68, 69, 70, 71], this mechanism is inherently error‐prone.

To address DNA damage (genotoxicity) and the lack of control associated with DSBs, researchers introduced precision genome‐editing tools in 2016. Komor et al. developed CBEs by fusing Cas9 nickases with cytidine deaminases, enabling C–G to T–A conversions without breaking both DNA strands [19, 72]. In parallel, the discovery of anti‐CRISPR proteins provided a means to regulate the timing and activity of these edits [73, 74, 75]. In 2017, further advances were achieved by tethering an engineered adenine (TadA) that evolved to generate ABEs, enabling A–T to G–C conversions [33, 76]. This line of research ultimately led to prime editing in 2019, a template‐directed method that uses a Cas9 nickase fused to reverse transcriptase. By employing a prime editing guide RNA (pegRNA), this system enables precise insertions, deletions, and all 12 possible base substitutions without requiring donor DNA [35, 77].

When the research focus moved beyond single‐nucleotide corrections toward inserting entire functional blocks of DNA, new genome writing tools were developed to handle these much larger sequences. In 2019, the discovery of CAST demonstrated that Tn7‐like elements co‐opt CRISPR components to directly insert multikilobase DNA segments into the genome [37, 38, 78]. Later, in 2021, the discovery of obligate mobile element‐guided activity (OMEGA) systems (IscB/TnpB) provided additional evolutionary clues, and it was found that IscB is likely an evolutionary ancestor of Cas9 [55, 79, 80]. After that, researchers combined prime editing with serine integrases to create tools like PASTE (2022), which made large‐scale insertions possible [39, 81]. More recently, between 2023 and 2024, the identification of bRNAs (IS110‐family insertion sequences) brought a new mechanism for DNA recombination. This system can achieve DNA insertion, sequence excision, and also structural inversions, relying on a mechanism that is largely distinct from standard CRISPR‐Cas platforms [40, 41, 82].

Finally, these technological advances have entered clinical testing and are increasingly incorporating computational design. In 2023–2024, the first CRISPR‐based therapies for sickle cell disease were approved, demonstrating the maturity of *ex vivo* editing [83, 84, 85, 86]. Concurrently, the field has entered an era of *de novo* design. Since 2025, generative AI models have begun producing synthetic editors and transposases with properties not found in nature [49, 50, 87]. The progression from simple nucleases in 2012 to AI‐designed synthetic platforms today provides the foundation for the strategies and mechanisms discussed in this review (Figure 1).

**Figure 1:**
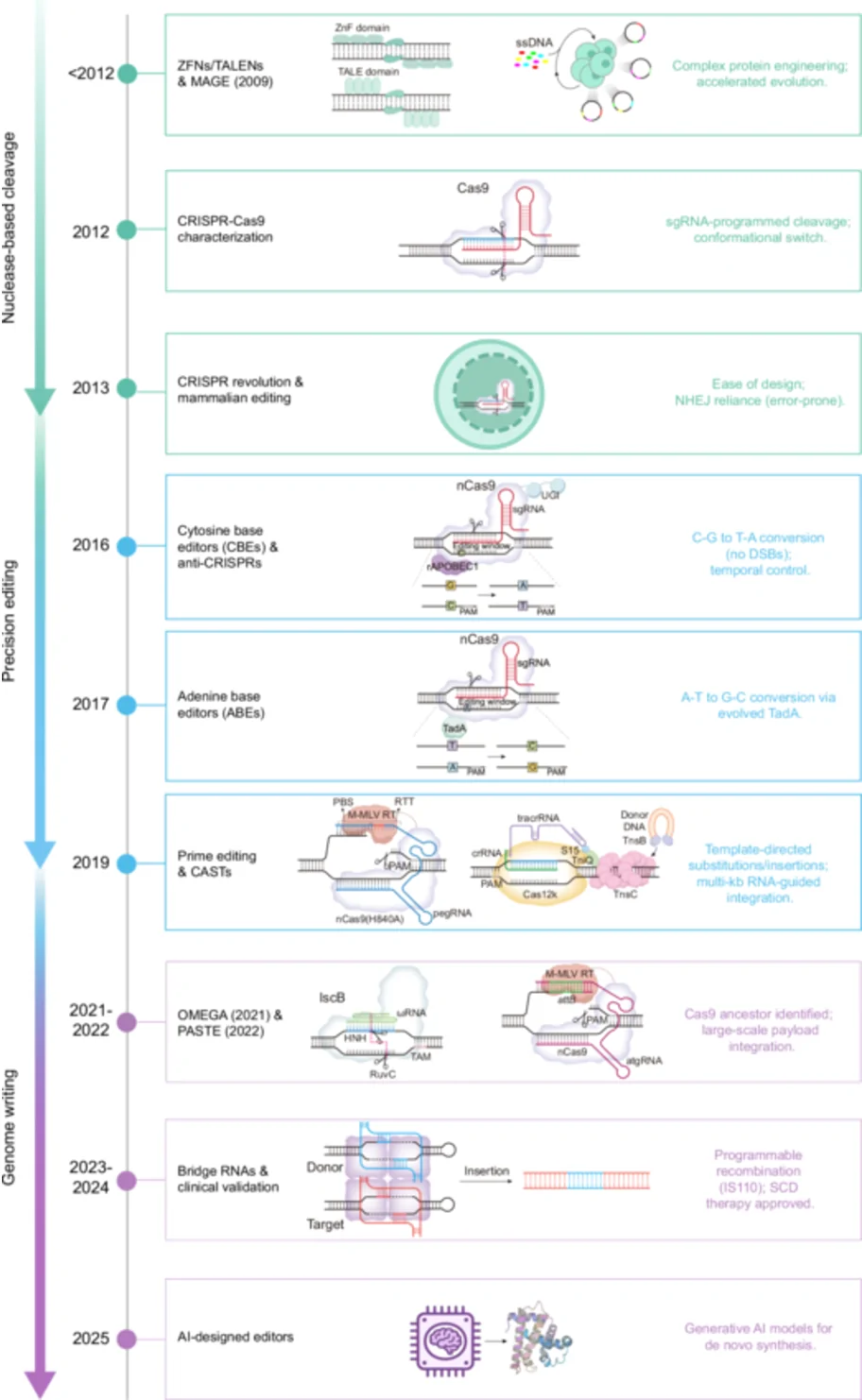
Evolutionary trajectory of genomic manipulation. Genome engineering has progressed through three main stages: nuclease‐based cleavage, precision editing, and genome writing. Early tools, such as zinc finger nucleases (ZFNs) and transcription activator‐like effector nucleases (TALENs) required complex protein engineering for each target, whereas multiplex automated genome engineering (MAGE) accelerated the process of evolution. In 2012, the characterization of the clustered regularly interspaced short palindromic repeats (CRISPR)‐Cas9 system showed how a single‐guide RNA (sgRNA) could program DNA cleavage through a conformational switch. When these tools were first applied in mammalian systems, they primarily relied on nonhomologous end joining (NHEJ) to disrupt target genes; however, this process is often error‐prone. Precision editing then appeared as a strategy to reduce the genotoxicity associated with double‐strand breaks (DSBs). Cytosine and adenine base editors use Cas9 nickases fused to cytidine deaminases or evolved tRNA adenine deaminase (TadA) deaminases to induce nucleotide changes without generating DSBs [19, 33]. Moreover, anti‐CRISPR proteins are used to regulate the timing of these activities. Prime editing uses a reverse transcriptase, enabling template‐directed DNA synthesis [35]. To overcome the limitations of cargo capacity, genome writing technologies were developed to support large, multikilobase integrations. These include RNA‐guided CRISPR‐associated transposases, obligate mobile element‐guided activity (OMEGA) systems that identify IscB as an evolutionary ancestor of Cas9, and programmable addition via site‐specific targeting elements (PASTE), which enables programmable payload insertion through integrase‐mediated recombination. Recent advances also include the discovery of IS110 bridge RNA systems for programmable recombination [40] and the clinical approval of CRISPR‐based therapies for sickle cell disease. Finally, generative artificial intelligence (AI) is being used to design synthetic effectors de novo, which helps make biological design more computationally autonomous [49].

### The paradigm shift from nuclease‐based disruption to synthetic writing

Overcoming the biological limitations associated with DSB repair has remained a major challenge for researchers in genome engineering. Early CRISPR technologies were primarily based on nuclease‐mediated disruption. In this stage, the induction of DSBs served as the central mechanism for genome modification, triggering the endogenous repair pathways, predominantly NHEJ [88, 89]. This approach proved effective for gene disruption, as NHEJ is error‐prone and frequently introduces random indels at the cut site [9, 16, 90]. As a result, this gene‐disruption mechanism has been widely applied in diverse contexts, including the generation of Warburg‐null metabolic states in mammalian cells [91, 92] and the improvement of rice resistance to bacterial blight through modification of pathogen‐binding motifs [91, 93]. However, the precision of these early systems was fundamentally limited because they relied heavily on stochastic repair outcomes. This makes them unsuitable for applications requiring exact sequence correction or seamless DNA insertion.

Besides the safety risks, we really need to move toward precision engineering to solve the problems of DSB‐based methods. Although HDR can theoretically enable precise edits through donor‐template‐guided repair, its efficiency is strongly limited by cellular biology. This limitation is particularly true in nondividing cells, where NHEJ is strongly favored over HDR [6, 94, 95]. Additionally, HDR primarily occurs during the S/G2 phases of the cell cycle, making it poorly suitable for treating tissues, such as the central nervous system [96, 97]. Another major concern is that DSBs involve serious genotoxic risks. Multiple studies have shown that Cas9‐induced breaks often lead to large on‐target deletions, chromosomal translocations, and other complex changes that standard PCR cannot detect [24, 25, 98, 99, 100]. Additionally, DSBs often act as hotspots where foreign DNA can insert itself. High frequencies of adeno‐associated virus (AAV) vector integration at these sites have raised concerns regarding insertional mutagenesis [101, 102, 103]. These structural abnormalities, together with p53‐mediated DNA damage responses, underscore the need for genome engineering tools that function without cutting the DNA backbone [104, 105, 106].

To address these challenges, research efforts have shifted to precision technologies like base and prime editing. Unlike earlier approaches, genome modification in these systems happens without relying on host repair pathways. In this context, precision primarily means the ability to control the repair results and avoid the generation of random indels. Base editors (BEs) achieve this effect by fusing deaminase domains to Cas9 nickases, which allows for the direct chemical conversion of bases, such as C–G to T–A. Overall, this method is quite efficient and leads to very few bystander effects [19, 107]. It has already shown promising outcomes in medical research. For example, in cardiovascular disease models, base editing corrected disease‐causing mutations with an efficiency of 70%, whereas the use of conventional nuclease‐based methods caused more toxicity [108, 109, 110, 111]. Prime editing, alternatively, relies on a write mechanism that involves a reverse transcriptase to insert new genetic information from an RNA template. This process, therefore, does not depend on the cell‐cycle constraints of HDR [112, 113, 114].

Recently, the most transformative advance is genome writing. This is a new method that goes beyond just fixing one spot and allows for much larger changes to the genome. Traditional genome editing is inherently an uncoupled, two‐step process of targeted cleavage followed by stochastic repair, whereas true genome writing directly couples DNA targeting with modification by synthesizing new DNA directly at the target site or seamlessly integrating large, ex vivo assembled DNA payloads [28, 115]. As a result, we can now think about adding multi‐kilobase instructions, whole metabolic pathways, or even programming new functional states. This shift is probably the best way to solve the cargo capacity problem that still limits base and prime editing to small changes. Emerging technologies have substantially expanded this capacity; for example, integrase‐mediated systems, such as PASTE, have demonstrated the ability to integrate payloads up to ~36 kb in dividing cells and maintain substantial multi‐kilobase insertional efficacy (up to 13.3 kb) in nondividing cells [39]. Concurrently, laboratory‐evolved CASTs have been shown to support the integration of multi‐kilobase payloads up to 15 kb in human cells [39, 116, 117]. Also, this new idea is more than just changing sequences; it involves writing epigenetic memory and 3D genomic structures [118]. For example, CRISPRoff can help us program gene states that are stable for a long time, whereas CRISPRa is used for strong but temporary activation [47, 119, 120]. Other systems can even change chromatin loops to rewire how genes are regulated [121, 122]. Taken together, all these advances show how we are moving away from just breaking genes and toward using the genome as a real substrate for complex biological design.

## 3 MECHANISMS OF NUCLEASE‐BASED EDITING AND REPAIR PATHWAY ENGINEERING

### Overview

Nuclease‐based genome editing operates at the intersection of programmable DNA recognition and endogenous cellular stress responses. The execution of this process relies on highly coordinated ribonucleoprotein structural rearrangements, conformational checkpoints, and kinetic competition among host DNA repair pathways, particularly NHEJ and HDR. As nuclease‐induced DSBs rely on stochastic endogenous repair mechanisms, editing precision remains limited and may lead to substantial structural genomic instability. Consequently, there is an evolutionary necessity to decode and synthetically modulate these cellular decision‐making networks. In this section, we review the structural mechanisms underlying target recognition and cleavage, delineate the dynamics that influence DNA repair pathway choice, and discuss strategies to bias repair outcomes, as well as the profound structural genomic instabilities triggered by these interventions.

### Structural mechanisms of target recognition and cleavage

Structural elucidation of RNA‐guided nucleases facilitated the transition from stochastic mutagenesis to precision genome engineering. Unlike ZFNs and TALENs, which rely on complex protein–DNA recognition codes [3, 4, 123, 124], Class 2 CRISPR‐Cas systems employ a ribonucleoprotein complex whose specificity is determined by Watson–Crick base pairing between a guide RNA and the target DNA. However, the mechanism extends far beyond simple hybridization; it involves a series of structural checkpoints, domain rearrangements, and kinetic proofreading steps that govern the transition from target search to phosphodiester bond hydrolysis.

The Type II effector Cas9 from *Streptococcus pyogenes* (SpCas9) consists of two primary parts: the recognition (REC) lobe and the nuclease (NUC) lobe. A positively charged central channel separates these two lobes, and this channel serves to hold the RNA‐DNA heteroduplex [6, 61, 125, 126]. Target recognition does not initiate directly with RNA‐DNA hybridization. Instead, the process begins through an interaction with the protospacer adjacent motif (PAM). For SpCas9, the common 5'‐NGG‐3' PAM binds to conserved arginine residues in the C‐terminal domain [127, 128, 129]. This specific interaction occurs at the major groove on the nontarget strand. Such binding leads to a local structural change known as the phosphate lock, in which a lysine/serine loop stabilizes the phosphate group just upstream of the PAM. This alteration creates a kink in the target strand, which lowers the energy required to unwind the DNA and helps the sgRNA to enter and form an R‐loop [128, 130, 131]. This mechanism may also explain why Cas9 is known to cause toxicity in high‐GC bacteria. The enzyme can bind to the PAM nonspecifically, even in the complete absence of the guide RNA [132, 133, 134, 135].

Following PAM binding, the seed region of the sgRNA plays the dominant role in determining specificity. Structural studies have shown that hybridization initially pauses after approximately 5 bp because of a steric gate formed by Tyr450. This gate must be displaced to allow continued R‐loop propagation [136, 137]. In addition, Cas9 employs a conformational checkpoint to maintain target fidelity high. The HNH nuclease domain remains catalytically inactive and positioned approximately 31 Å away from the cleavage site until the R‐loop is fully formed. Once hybridization is complete, the noncatalytic REC3 domain senses the extended heteroduplex and transmits a signal that rotates the REC2 domain and repositions the HNH domain into the active cleavage conformation [136, 138, 139]. If mismatches are present, particularly in PAM‐distal regions, the whole complex becomes trapped in a linear inactive state. This prevents HNH activation and blocks DNA cleavage [140, 141, 142]. Once activated, Cas9 uses the HNH domain to cleave the target strand and the RuvC domain to cleave the nontarget strand, generating a blunt‐ended DSB approximately 3 bp before PAM [64, 143, 144].

The Type V system Cas12a (Cpf1) is quite different from Cas9 in its cleavage mechanism. It has a unique structure that relies on only one RuvC domain, and it produces staggered DNA breaks. Cas12a recognizes a T‐rich PAM (5′‐TTTV‐3′). Before target binding, it shapes the seed sequence into an A‐form helix, which may help to lower the energy cost [145, 146, 147]. The wedge (WED) domain also plays a major role in unwinding the DNA and recognizing the PAM [148, 149]. Regarding the mechanism, Cas12a produces DSBs with 5‐nucleotide 5' overhangs in several steps. The RuvC domain cuts the nontarget strand first because it is easier to access. After this, a structural change puts the target strand into the same site for cleavage [145, 146, 150](Figure 2A). This distal cleavage leaves the PAM and seed sequence intact after NHEJ repair. This makes repeated cleavage cycles possible, and these cycles can increase HDR efficiency or lead to large deletions [151, 152, 153]. Also, evolutionary studies recently found hypercompact variants like Cas12f. This enzyme works as an asymmetric homodimer with a sgRNA. This finding highlights the structural diversity in CRISPR‐Cas systems [154, 155, 156, 157]. Some new Type I variants, like Cas8‐HNH and Cas5‐HNH, can make precise DNA overhangs without damaging the DNA the way Cas3 normally does [158, 159, 160, 161]. Furthermore, researchers discovered pro‐CRISPR factors like PcrIIC1. This shows that accessory proteins might affect the activity of Cas effectors, which can improve binding and change PAM recognition [162, 163].

**Figure 2:**
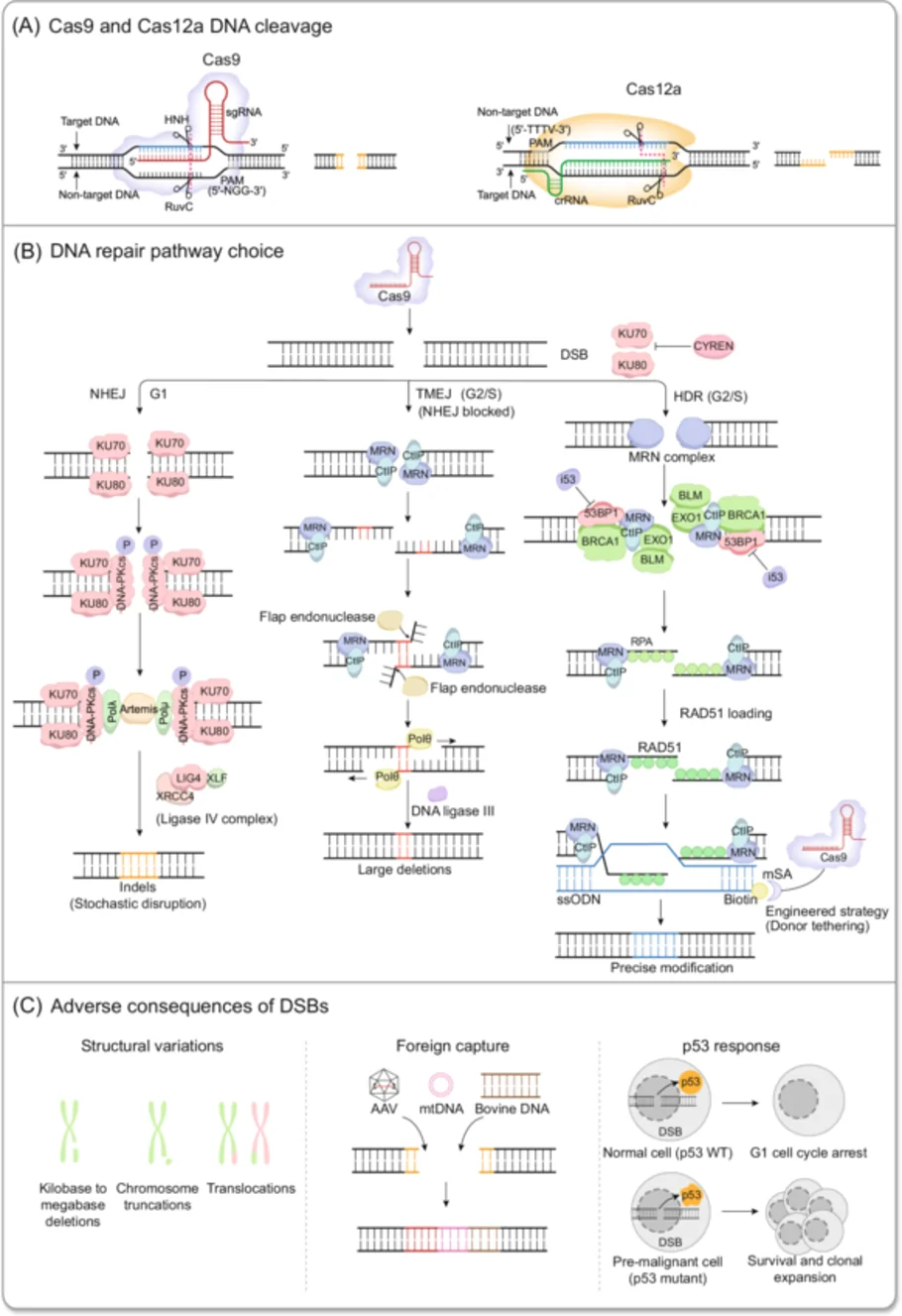
Mechanisms of clustered regularly interspaced short palindromic repeats‐CRISPR‐associated (CRISPR‐Cas) nucleases, DNA repair pathway competition, and associated genotoxicities. (A) Structural mechanisms of target cleavage. SpCas9 cleaves target DNA via paired HNH and RuvC domains to generate blunt double‐strand breaks (DSBs) near a 5'‐NGG‐3' protospacer adjacent motif (PAM) [128]. By contrast, Cas12a utilizes a single RuvC domain to produce staggered DSBs, leaving 5' overhangs located distal to the 5'‐TTTV‐3' PAM [146]. (B) Selection of endogenous repair pathways and strategies to bias repair outcomes. Cellular resolution of DSBs is largely dictated by pathway competition across different cell cycle phases. In the G1 phase, the 53BP1‐Shieldin axis acts alongside Ku70/80 to drive canonical nonhomologous end joining (NHEJ), a process that typically results in the formation of stochastic indels [88]. As cells transition into the S and G2 phases, CYREN suppresses NHEJ. Concurrently, BRCA1 initiates homology‐directed repair (HDR) by promoting extensive DNA end resection, which requires the involvement of the MRN complex, CtIP, EXO1, and BLM. Several approaches have been developed to bias the cellular repair machinery toward precise HDR for therapeutic applications. For instance, the engineered i53 variant competitively blocks 53BP1, alleviating the barrier to DNA end resection [89]. Alternative methods rely on spatial tethering. Pairing Cas9‐mSA with biotinylated single‐stranded oligodeoxynucleotides (ssODNs), for example, increases the local concentration of donor templates directly at the cleavage site. In some instances, resected DNA ends undergo theta‐mediated end joining (TMEJ) dependent on polymerase θ (Pol θ). This acts as a mutagenic backup pathway and is frequently associated with large genomic deletions. (C) Structural genomic instability and cellular stress responses. The introduction of targeted DSBs can occasionally induce severe structural damage to the genome. Such anomalies range from kilobase‐ to megabase‐scale deletions, as well as chromosomal truncations and translocations. The induced breaks may also inadvertently capture exogenous DNA, including sequences derived from adeno‐associated virus (AAV) vectors, mitochondria, or bovine sources [25]. Furthermore, the generation of DSBs triggers a robust p53‐dependent DNA damage response. In wild‐type cells, this leads to cell cycle arrest at the G1 phase. However, this biological response inadvertently establishes a selective pressure that favors the survival and expansion of premalignant, p53‐mutant cell populations.

The limitations of natural nucleases have been addressed through rational protein engineering to expand the targeting range and improve specificity [121]. The strict need for NGG in SpCas9 was changed in variants, such as SpRY. Researchers achieved this by removing steric clashes with certain bases and introducing additional interactions with the DNA backbone, enabling near‐PAM‐independent targeting [164, 165, 166, 167]. For high‐fidelity variants, such as HypaCas9, modifications to the REC3 domain increase the stringency of the conformational checkpoint, ensuring that near‐perfect guide–target pairing is required before activation of the HNH domain [138, 168, 169]. Similarly, the SuperFi‐Cas9 variant targets the RuvC loop to destabilize the active state in the presence of mismatches, thereby preventing off‐target cleavage [141, 170]. These structural insights highlight that the transition from natural bacterial systems to precise genome engineering tools depends on the rotational modification of protein domains and RNA scaffolds [171, 172, 173, 174, 175].

### Mechanisms of DNA repair pathway choice

In most cases, nuclease‐based genome engineering depends on how the cell resolves targeted DNA damage. Whether the result is stochastic or precise is determined by the interplay between DNA repair pathways rather than the nuclease itself. This balance is driven by a strict competition between NHEJ and HDR, which is mostly controlled by DNA end resection—the process by which the 5'‐strand is degraded to generate 3'‐single‐stranded DNA (ssDNA) overhangs.

The endogenous repair pathway choice is primarily determined by whether DNA ends become accessible to the resection machinery. In the absence of a repair template, or during the G1 phase of the cell cycle, repair typically favors NHEJ. This state is enforced by 53BP1, a master regulator that recognizes checkpoints, such as H2AK15ub and H4K20me2 at the damage sites [89, 176]. Upon binding, 53BP1 recruits replication timing regulatory factor 1 (RIF1), followed by the Shieldin complex, which includes SHLD1, SHLD2, SHLD3, and REV7. Structural studies have shown that the SHLD2 subunit uses its OB‐fold domains to bind ssDNA, physically blocking access by nucleases and forcing the cell toward blunt‐end ligation [88, 177]. This pathway is epistatic; although 53BP1 can independently reorganize chromatin, its capacity to suppress resection depends entirely on the RIF1–Shieldin axis [178, 179, 180].

To initiate the HDR pathway for precision editing, the cell must overcome this anti‐resection barrier. BRCA1 and its partner BARD1 are the primary factors opposing the 53BP1–Shieldin axis. Interestingly, BRCA1–BARD1 acts as a direct helper for the resection machinery, rather than acting as a passive inhibitor of 53BP1. This complex interacts with and helps long‐range resection nucleases, such as EXO1 and the BLM–DNA2 complex, to work more effectively [181, 182]. The balance between these opposing forces is also affected by factors, such as DYNLL1, which stops resection by interacting with MRE11. Loss of DYNLL1 can restore HDR competency even in the absence of BRCA1 [183, 184]. Ultimately, the repair pathway choice reflects a dynamic balance between end‐protection factors (53BP1–Shieldin) and those that promote end resection to enable strand invasion (BRCA1–CtIP–MRE11).

In addition to these factors, this competition is strictly linked to the cell cycle and metabolic state. For example, when sister chromatids are available as templates, HDR primarily occurs during the S and G2 phases of the cell cycle. This restriction is enforced by cyclin‐dependent kinases (such as CDK1 and CDK2), which phosphorylate resection factors, such as CtIP. At the same time, CDK12 maintains the baseline expression of important homologous recombination (HR) genes, including ATM, BRCA1, and FANCD2. Loss of CDK12 results in a BRCAness phenotype and a lack of HR [185, 186, 187]. Furthermore, the cell‐cycle regulator of NHEJ (CYREN), a cell‐cycle‐dependent inhibitor, binds the Ku70/80 heterodimer during S and G2 phases to protect resected overhangs from being processed by NHEJ, which helps promote HDR [188]. Metabolic signals also influence this decision. For instance, the accumulation of lactate (typical of the Warburg effect) helps HR by inducing lactylation of MRE11 and NBS1, which strengthens the MRN complex [189, 190]. However, metabolites, such as 2‐hydroxyglutarate, can suppress KDM4B activity and hide the histone signals needed to initiate HDR [191, 192].

When the usual NHEJ and HDR pathways are disrupted, or when certain DNA substrates are present, cells may rely on alternative repair mechanisms. These methods often leave distinct mutational signatures. Microhomology‐mediated end joining (MMEJ), also known as theta‐mediated end joining (TMEJ), is one such alternative to NHEJ. This pathway depends on Pol θ (POLQ) and is highly harmful because it generates deletions based on microhomologies [62]. This competition becomes particularly evident when the usual NHEJ is blocked, for example, through the loss of LIG4. In such cases, cells are forced to rely on TMEJ, resulting in frequent large deletions [100, 193, 194]. Despite these risks, local sequence can influence TMEJ outcomes, which may help predict editing results [16, 90, 195]. Additionally, single‐stranded template repair (SSTR), commonly used during editing with single‐stranded oligodeoxynucleotide (ssODN) donors, operates differently from plasmid‐based HDR. SSTR requires the helicase HELQ to displace replication protein A (RPA) from the ssDNA, allowing strand annealing, and depends heavily on the Fanconi anemia pathway [190, 196, 197, 198].

The actual structure of the DNA break also plays a key role in recruiting repair factors. For instance, SpCas9 cuts DNA to leave blunt ends. The Ku70/Ku80 heterodimer recognizes these ends very quickly and protects them from resection, which naturally pushes the repair process toward NHEJ [80, 199]. Cas12a, on the other hand, leaves staggered ends with 5' overhangs. Based on in vitro cleavage assays using plasmid or short double‐stranded DNA substrates along with Sanger sequencing, early observations showed that these overhangs usually span 4–5 nucleotides [147, 200]. Later biochemical tests, relying on urea polyacrylamide gel electrophoresis and high‐throughput nanopore sequencing, found that this length actually varies quite a bit and depends heavily on the crRNA spacer length. When standard spacers (≥20 nucleotides) are used, the breaks mostly have 5‐nucleotide overhangs. But truncated spacers (16–19 nucleotides) can shift the cut site on the non‐target strand, and this shift leads to longer 5' overhangs that reach 6–8 nucleotides [201, 202].

Even with this structural variation, NHEJ can still process these staggered ends. When this happens, the precise repair often leaves the PAM sequence intact, which means the site might undergo repeated rounds of cleavage [203, 204]. It is also worth noting that non‐DSB tools like base and prime editors might inadvertently trigger these same pathways. Sometimes, the nicking intermediates turn into actual DSBs and are repaired by TMEJ. This process can cause large deletions that are rare but still a serious concern [100, 205]. Because the cell's repair machinery responds directly to these structural differences, understanding such endogenous dynamics provides a necessary basis for the engineering strategies discussed in the next section.

### Strategies for biasing repair outcomes

To achieve more predictable outcomes in precision genome engineering, researchers have developed multiple strategies to override the cell's natural preference for NHEJ and redirect repair toward HDR. The most direct approach involves manipulating the repair process through chemical inhibition or genetic engineering [206, 207]. For example, inhibition of key NHEJ factors, such as DNA ligase IV (using SCR7 or Ad4) or DNA‐PKcs (DNA‐dependent protein kinase catalytic subunit using small molecules, such as NU7441 and M3814) blocks the usual ligation pathways. This can substantially improve HDR efficiency, in some cases by up to 19‐fold [16, 208, 209, 210, 211, 212]. However, simply suppressing NHEJ does not always guarantee precise repair, because cells may instead activate the highly mutagenic MMEJ pathway. Therefore, achieving high‐fidelity repair often requires a combined strategy involving simultaneous inhibition of DNA‐PKcs and Pol θ. This suppresses NHEJ and MMEJ, thereby directing the cell toward HDR [193, 213, 214]. Another effective strategy involves altering the balance between 53BP1 and BRCA1. The engineered ubiquitin variant i53 competitively inhibits 53BP1 recruitment to chromatin [89, 145, 215], thereby promoting DNA end resection and substantially increasing HDR efficiency [58, 216, 217, 218] (Figure 2B).

Beyond pathway inhibition, the design and localization of donor templates are also critical for improving editing precision. ssODNs are commonly used for small edits and function through a synthesis‐dependent strand annealing mechanism that is distinct from plasmid‐based repair. These ssODNs are generally more effective when designed to match the nontarget strand, which facilitates faster annealing after DNA cleavage [60, 196, 219]. To address the problem of donor–template diffusion, tethering strategies have been developed to localize donor DNA directly at the break site. Examples include systems, such as Cas9‐mSA, which recruits biotin‐labeled donor templates [220], and retron‐based systems, such as CRISPEY, which directly link donor DNA to the sgRNA. These strategies ensure that the repair instructions arrive simultaneously with nuclease activity [221, 222, 223, 224]. Moreover, reducing RNase H activity allows endogenous RNA molecules to function as repair templates by stabilizing RNA–DNA hybrids needed for repair [225, 226, 227].

As HDR occurs predominantly during the S and G2 phases of the cell cycle, spatiotemporal control is required. Using Cas9 as a ribonucleoprotein (RNP) complex makes it active for only a short time; this cuts down on off‐target effects and helps the timing match the best repair window [62, 228, 229]. For medical use, we can fuse Cas9 to a Geminin degron so the nuclease stays stable only during the S/G2 phase, when the HDR machinery is actually available [230, 231]. When editing embryos (germline), the timing is very important to prevent mosaicism (where only some cells are edited). We have found that injecting tools during the oocyte's M phase, rather than the zygote's S phase, promotes repair using the maternal chromosome as a high‐quality template to correct paternal mutations [232, 233, 234].

Alternative strategies have also been developed to use NHEJ for inserting genes because HDR is inefficient in nondividing cells, such as neurons. In homology‐independent targeted integration (HITI) [235], donor DNA is flanked by Cas9 recognition sites so that, once cleaved, it can be directly ligated into the genome through NHEJ [236, 237]. If the donor is inserted in the wrong orientation, the sites remain intact and allow repeated cleavage, creating a thermodynamic bias that ultimately favors correct insertion. Furthermore, homology‐mediated end joining (HMEJ) combines features of both approaches, with CRISPR target sites positioned adjacent to long homology arms. This strategy leverages the specificity of HDR and the efficiency of end joining, enabling targeted gene insertion even in nondividing cells where HDR is typically inefficient [83, 238]. Overall, these approaches illustrate a shift in the field from attempting to overcome endogenous DNA repair pathways to strategically harnessing them for improved genome‐engineering outcomes [239, 240].

### Structural genomic instability and cellular response

The induction of targeted DSBs is the fundamental step of nuclease‐based editing and can activate a complex cellular DNA damage response that extends far beyond the intended formation of small indels. Although early validation methods focused on short‐range PCR amplification, recent high‐resolution genomic analyses have shown that DSBs can lead to severe structural genomic instability. These unintended outcomes range from kilobase‐scale deletions to whole‐chromosome loss, indicating that Cas9‐induced damage affects regions beyond the immediate cleavage site. In addition, the cellular response to such breaks exerts strong selective pressure, which may alter the clonal composition of edited populations and raise important safety concerns for therapeutic applications (Figure 2C).

Unlike the simple localized repair model, Cas9‐induced DSBs can actually destabilize the broader chromosomal architecture. This disruption is largely driven by DNA end resection and alternative end‐joining pathways. According to recent long‐read sequencing and phenotypic screening studies, these breaks frequently lead to large deletions and chromosomal truncations, and such structural changes can sometimes extend for megabases from the initial cut site all the way to the telomere [7, 24, 194] The exact mechanism may be complex, but it appears that polymerase theta (Pol θ)‐dependent end joining (TMEJ) plays a key role in mediating these large deletions after the DNA ends are extensively resected. Interestingly, when researchers inhibit Pol θ or the ataxia telangiectasia and Rad3‐related protein kinase (ATR), the occurrence of these massive deletions is reduced, while the formation of small indels remains relatively stable [100, 205]. This phenomenon introduces a serious proximity bias during functional screens. Because of the large‐scale deletions, specific CRISPR knockouts may unexpectedly show phenotypes that actually come from the loss of neighboring genes. In addition, this effect becomes much worse when apoptotic pathways are suppressed [106], which allows the cells with truncated chromosome arms to survive and proliferate. Of course, the helicase‐nuclease Cas3 intentionally induces long‐range unidirectional deletions to achieve gene ablation [161, 241]. However, when Cas9 generates these large structural variants in a stochastic way, it poses a substantial genotoxic risk to the cells.

In addition to linear deletions, DSBs can trigger catastrophic chromosomal rearrangements. On‐target cleavage has been shown to cause chromothripsis, a process in which the formation of an acentric chromosome fragment leads to micronucleation, rupture, and random reassembly of the shattered chromosome arm [23]. Simultaneous breaks on different chromosomes during multiplex editing further increase the risk of translocations. Live‐cell imaging has shown that pairing of DSB loci can induce translocations in chimeric antigen receptor T (CAR‐T) cells targeting the T‐cell receptor (TCR) alpha constant region and CD52 [242, 243, 244]. Even single‐locus targeting may produce complex rearrangements, as demonstrated by extensive pericentric inversions [245, 246, 247] resulting from variant‐specific off‐target cleavage. Furthermore, the repair machinery may fail entirely in specific biological contexts, such as human embryos and T cells, leading to segmental aneuploidy or loss of the entire targeted chromosome [98, 248].

The tendency of DSBs to capture exogenous DNA further compounds structural genomic instability. When breaks are repaired in the presence of viral vectors, such as AAV, high‐frequency integration of viral inverted terminal repeat (ITR) sequences can occur via NHEJ. In neuronal editing, AAV capture rates have been observed to approach 50%, and the resulting concatemers are often undetectable by standard genotyping, posing a risk of insertional mutagenesis [101, 102, 103, 249]. This capture mechanism extends beyond viral vectors to include mitochondrial DNA and bovine DNA from culture media, a process suppressed by the factors DNA2 and MCM10 [250, 251]. These findings indicate that DSBs function as genomic integration hotspots for foreign genetic material, thereby compromising the safety of in vivo editing strategies.

The p53‐mediated cellular response is an important factor in nuclease‐based editing. DSBs trigger a robust p53‐dependent G1 cell‐cycle arrest, which can paradoxically inhibit the S/G2‐dependent HDR pathway necessary for precise genome correction [20, 252]. Moreover, this response creates an important risk factor that provides a strong selective pressure to support the survival and expansion of p53‐deficient cells. In competitive assays, TP53‐mutant clones, which are often p53‐deficient, show a clear growth advantage under Cas9‐induced stress, suggesting that therapeutic editing may inadvertently enrich premalignant populations [104]. This risk is further exacerbated when targeting copy‐number‐amplified regions, where excessive DNA damage includes a lethal response independent of gene function [21, 253]. Thus, structural and cellular limitations, including genotoxicity, chromosomal instability, and p53‐mediated selection, represent major safety challenges for nearly all DSB‐based methods. Therefore, the transition to non‐DSB‐based precision technologies is expected to become increasingly necessary. These alternative approaches are discussed in the next section.

### Section summary

In practice, the final results of nuclease‐based editing depend heavily on the kinetic competition between exogenous enzymatic cleavage and the endogenous DNA repair hierarchies of the host. Targeted DNA cleavage often leads to the intended genetic changes. However, the genotoxicity caused by DSBs still poses a major hurdle for high‐fidelity in vivo therapeutic applications. For example, DSBs may cause severe damage, such as chromosomal truncations and chromothripsis, and they also increase the risk of integrating exogenous viral DNA. To deal with these problems, future strategies might need to combine transient pathway inhibitors and spatiotemporal cell‐cycle synchronization, along with the use of novel biomaterials. These approaches are expected to restrict nuclease activity, which could subsequently limit the downstream damage cascades. However, the optimization of DSB‐mediated editing seems to be reaching its biological limits. Because of this, the field is currently shifting toward new strategies that separate genome editing from the endogenous repair pathways. The underlying mechanisms involved may be quite complex. Therefore, the following chapter will focus on precision editing methods that rely on direct chemical and enzymatic modifications.

## 4 PRECISION ERA: BASE, PRIME, AND EPIGENETIC EDITING

### Overview

Base, prime, and epigenetic editing represent major advances in precision genome engineering. These tools have transformed the field by shifting genome editing away from simple DNA cleavage toward precise nucleotide modification through direct chemical conversion and template synthesis. These systems generally rely on impaired Cas proteins that no longer generate DSBs. Instead, these Cas proteins function as programmable targeting platforms fused to specialized effector domains, such as single‐strand‐specific deaminases, engineered reverse transcriptases, or epigenetic modifiers. Structured RNA complexes then guide the entire system to the desired genomic locus. As these approaches avoid the harmful DSBs associated with conventional nucleases, they are not constrained by the cell‐cycle dependence of HDR. This provides substantially greater control over editing outcomes. In particular, this improved precision enables the safer correction of disease‐causing point mutations and allows modulation of regulatory elements, even in nondividing cells, such as postmitotic tissues. In this section, we examine the mechanisms of base editing and recent improvements in prime editing. We also briefly discuss the fundamental principles of epigenetic editing and transcriptional regulation, followed by emerging strategies for engineering organelle genomes.

### Deaminase‐mediated base editing

Base editors (BEs) are genome‐modification tools that circumvent the need for DSBs and exogenous repair templates. By fusing CRISPR‐Cas nickases to single‐strand–specific deaminases, BEs catalyze the direct chemical conversion of nucleobases within a programmable editing window, thereby avoiding the genotoxicity and indel formation typically associated with Cas9‐mediated double‐strand cleavage [254, 255, 256]. This paradigm shift began with the development of CBEs, which facilitate C–G to T–A transitions. The pioneering molecule, BE3, was engineered by combining rat apolipoprotein B mRNA‐editing enzyme catalytic subunit 1 (APOBEC1) cytidine deaminase with Cas9 nickase (nCas9) and a uracil glycosylase inhibitor (UGI). Mechanistically, the deaminase converts C to U on the single‐stranded nontarget DNA loop formed by the Cas9 R‐loop complex. The UGI component prevents the excision of the uracil intermediate by endogenous uracil DNA glycosylase, while nCas9 introduces a nick in the opposing strand. This nicking event guides the cellular mismatch repair machinery to resolve the U–G mismatch in favor of uracil, ultimately resulting in permanent conversion of the targeted C–G pair to a T–A pair after DNA replication [19, 72, 257, 258] (Figure 3A).

**Figure 3:**
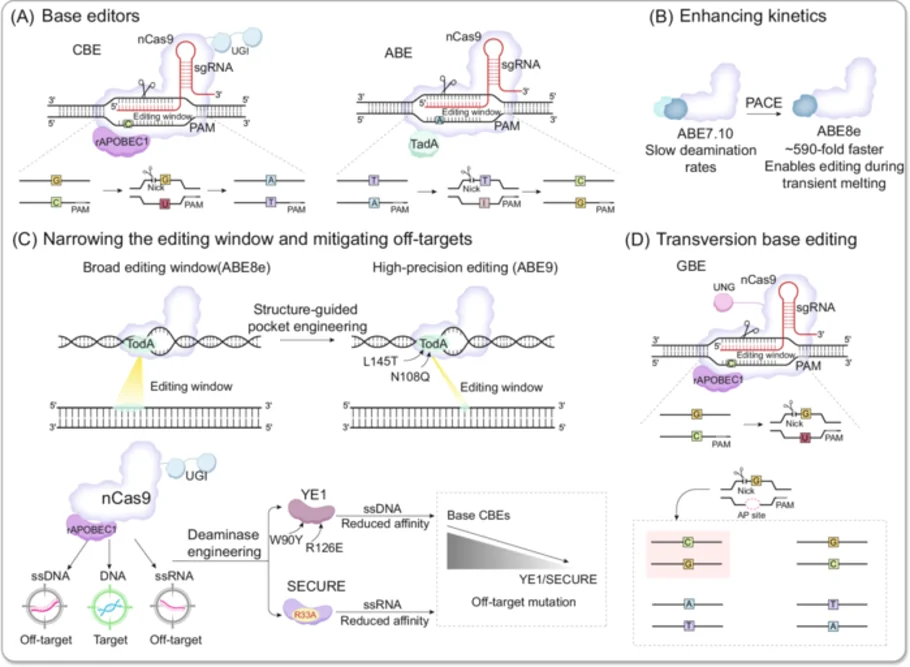
Mechanisms and engineering of deaminase‐mediated base editors. (A) Canonical editors. Cytosine base editors (CBEs) fuse a Cas9 nickase (nCas9) to a cytidine deaminase (rat apolipoprotein B mRNA editing enzyme, catalytic polypeptide 1 (rAPOBEC1)) and a uracil glycosylase inhibitor (UGI), catalyzing targeted C‐to‐U deamination [19]. Adenine base editors (ABEs) tether an engineered tRNA adenine deaminase (TadA) to nCas9, converting adenosine to inosine [33]. Subsequent nicking of the nonedited strand directs cellular mismatch repair, yielding C–G to T–A and A–T to G–C transitions, respectively. (B) Kinetic enhancement. Phage‐assisted continuous evolution (PACE) generated ABE8e, exhibiting an approximately 590‐fold increased deamination rate compared to ABE7.10, facilitating efficient deamination during transient DNA melting [259]. (C) Precision engineering. Structure‐guided mutations (N108Q/L145T) in the ABE9 catalytic pocket sterically restrict substrate access, narrowing the editing window and reducing unintended adjacent mutations [255]. Furthermore, rationally engineered cytosine base editor variants, such as YE1 (W90Y/R126E) and SECURE (R33A) demonstrate reduced intrinsic affinity for single‐stranded DNA and RNA, thereby minimizing Cas‐independent off‐target activity. (D) Transversion editing. Glycosylase base editors (GBEs) incorporate uracil DNA glycosylase (UNG) to actively excise the deaminated uracil intermediate, generating an apurinic/apyrimidinic (AP) site [135]. Translesion synthesis pathways then resolve this abasic site to yield targeted C–G to G–C or C–G to A–T transversions.

While CBEs depend on naturally occurring enzymes, researchers had to build ABEs from the ground up, as no natural enzyme was known to deaminate adenine in DNA. Through seven rounds of directed evolution on the *Escherichia coli* tRNA adenine deaminase (TadA), the enzyme was modified to recognize ssDNA, and this led to the generation of the ABE7.10 variant. This system changes adenosine to inosine. DNA polymerases then interpret the inosine as guanine, and this process leads to A–T to G–C transitions [33, 260]. Unlike CBEs, ABEs do not require the inhibition of glycosylase. A major reason is that the endogenous repair machinery does not easily remove inosine. Consequently, ABEs show a very high product purity (>99.9%) and almost no indel formation [33, 261]. Early versions showed a relatively slow catalytic rate. To address this problem, phage‐assisted continuous evolution (PACE) was applied to develop ABE8e, a variant that shows a deamination rate about 590 times faster than ABE7.10. Based on structural studies, ABE8e appears to stabilize the target DNA in a tRNA‐like conformation. This conformational change helps adenine deamination happen during transient DNA melting, even before the full formation of the R‐loop, which is shown in Figure 3A,B [262, 263, 264]. Also, these systems can work together with multiple Cas proteins, such as Cas12a and Cas9 variants, that have relaxed PAM requirements, which expands the target space for editing [265]. However, it is still quite important to monitor off‐target effects [266, 267, 268].

One limitation of BEs is bystander editing. This phenomenon involves unintended modifications at non‐target C or A bases that are located within the editing window. To improve the precision of editing, researchers have developed several variants with narrower editing windows [269, 270, 271, 272]. For example, ABE9 variants contain specific mutations like TadA‐8e N108Q/L145T. These mutations limit the editing activity to just one or two bases. This structural change reduces bystander editing by over 300‐fold when compared to ABE8e [255, 273, 274, 275] (Figure 3C). In a similar way, high‐fidelity CBEs that use eA3A are able to discriminate between target and bystander C bases, and they do this by recognizing specific TCR motifs [276, 277]. Besides these local issues, early BEs also showed many Cas‐independent off‐target effects. These editors often caused random base changes across the genome and the transcriptome. Researchers tried to solve this problem by modifying the deaminase domains to lower their intrinsic affinity for DNA and RNA. For example, variants like the YE1 and SECURE editors lead to a significant decrease in these off‐target mutations, while their on‐target editing power remains relatively stable [243, 278, 279, 280] (Figure 3C).

Base editing has also been expanded to enable transversion mutations, overcoming the limitation of earlier systems that could perform only transition edits. Glycosylase base editors (GBEs) use the repair pathway that CBEs usually try to block. GBEs link Cas9 to a deaminase and uracil DNA glycosylase (UNG). This removes the deaminated base and generates an abasic site. Then, translesion synthesis (TLS) polymerase REV1 inserts deoxycytidine monophosphate (dCMP) opposite the apurinic/apyrimidinic (AP) site, and Polζ extends the product, helping fix the C:G‐to‐G:C outcome [135, 281] (Figure 3D). Furthermore, combining these enzymes with Cas9 variants like SpRY and SpG means almost the whole genome can now be edited [32, 34, 167, 282]. Together, these advances have transformed base editing from a simple tool into a very useful platform for correcting mutations [31, 32, 283, 284, 285]. This progress also gave a good foundation for the writing strategies used in prime editing.

### Prime‐editing mechanism and optimization

Although BEs represent a substantial advancement in precision by enabling chemical transition mutations, they cannot generate transversion mutations or precise indels. Prime editing (PE), developed in 2019, addresses these limitations by employing a template‐directed DNA synthesis approach capable of installing all 12 possible base‐to‐base conversions, as well as small structural alterations, without requiring DSBs or donor DNA [286, 287, 288]. The prime editor PE2 comprises a Cas9 H840A nickase fused to an engineered Moloney murine leukemia virus (M‐MLV) reverse transcriptase (RT). Target specificity and genetic instructions are encoded within a pegRNA, a bifunctional molecule that extends the standard sgRNA with a 3' extension containing a primer‐binding site (PBS) and a reverse transcription template [35, 77, 289] (Figure 4).

**Figure 4:**
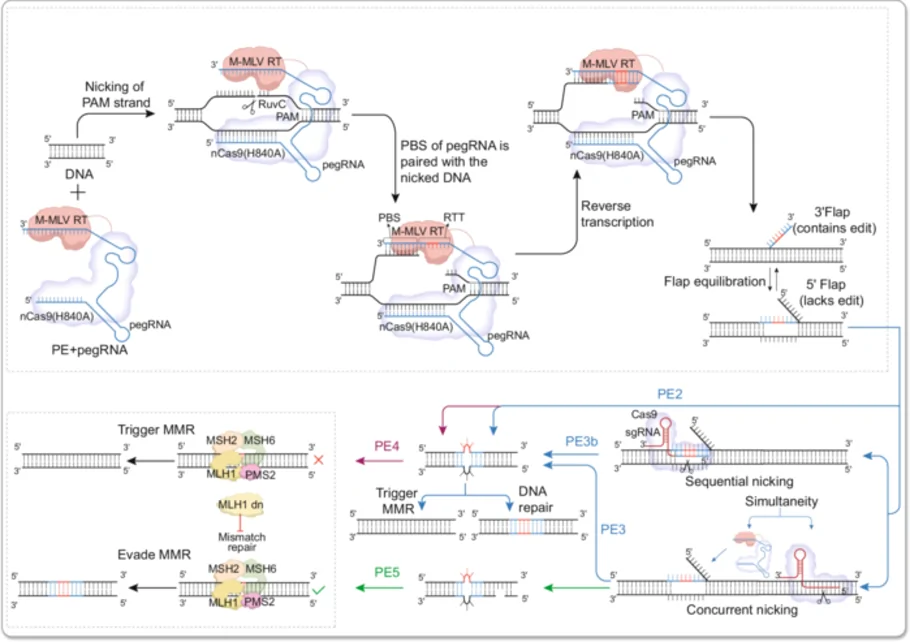
Mechanism and optimization of prime editing. The prime editor 2 (PE2), which has a Cas9(H840A) nickase and an engineered Moloney murine leukemia virus (M‐MLV) reverse transcriptase (RT), goes to the target site with a prime editing guide RNA (pegRNA) [35]. After binding, the nickase cleaves the protospacer adjacent motif (PAM)‐containing DNA strand. The resulting 3' DNA flap then pairs with the primer‐binding site (PBS) of the pegRNA, and the RT starts to write the edit using the reverse transcription template (RTT). This makes an intermediate with an edited 3' flap and an unedited 5' flap. Then, the flaps shift and enzymes like flap endonuclease 1 (FEN1) remove the unedited 5' flap, allowing ligation of the edited strand and formation of a DNA heteroduplex. To help the cell choose the edited strand, the PE3 method uses an additional nicking sgRNA (nsgRNA) to also nick the other strand. Another way is PE3b, which uses sequential nicking to avoid double‐strand breaks (DSBs). The mismatch repair (MMR) pathway, through MutSα (MSH2/MSH6) and MutLα (MLH1/PMS2), often detects the heteroduplex and changes it back to the wild type [288]. To address this, PE4 and PE5 systems express a dominant‐negative MLH1 (MLH1dn) to suppress MMR and ensure edit incorporation [287]

PE involves sophisticated coordination among DNA nicking, hybridization, and polymerization. The Cas9 nickase cleaves the PAM‐containing strand upon binding the target locus. The resultant 3' DNA flap hybridizes with the PBS of the pegRNA, priming the fused RT to synthesize DNA using the reverse transcription template. This process generates a branched intermediate containing 3' and 5' flaps with the edited and original sequences, respectively. Through flap equilibration and the excision of the unedited 5′ flap, likely mediated by endogenous 5' exonucleases, such as flap endonuclease 1 (FEN1) [76, 290], the edited 3' flap is ligated into the genome, creating a DNA heteroduplex, as shown in Figure 4. To bias repair of the heteroduplex [35] toward the desired edit, the PE3 strategy introduces a second standard sgRNA to nick the non‐edited strand. This nicking biases the cellular repair machinery to use the newly edited strand as the template for repair, increasing the efficiency by 1.5‐ to 4.2‐fold compared with that of PE2. The PE3b strategy further refines this approach by inducing the second nick only after the desired edit has been installed, thereby significantly reducing the risk of indel formation associated with simultaneous dual nicking [35, 150] (Figure 4).

Although PE has broad applications, the cellular mismatch repair (MMR) pathway often blocks its function. Specifically, MMR machinery, such as the MutSα (MSH2/MSH6) and MutLα (MLH1/PMS2) complexes can recognize the edit‐containing heteroduplex as a replication error. It prefers to remove the newly synthesized strand, thereby reverting the sequence back to the wild type [287, 288, 291]. To address this problem, researchers developed the PE4 and PE5 systems, which temporarily inhibit MMR through coexpression of a dominant‐negative MLH1 (MLH1dn). By suppressing the pathway globally, these systems increased the editing efficiency by an average of 7.7‐fold (Figure 4) [287]. However, systemic MMR suppression may lead to complex oncogenic risks. Therefore, recent studies have used generative AI tools, including AlphaFold3 and RFdiffusion, to design compact peptide binders. These binders can disrupt specific MMR protein interfaces. For example, when these small binders are fused to the editor (e.g., PE7–SB2), the editing efficiency is substantially improved while avoiding the toxicity associated with global pathway inhibition [125, 292]. Moreover, introducing silent mutations near the intended edit can create a mismatch bubble that evades MMR recognition and shifts repair outcomes toward the desired sequence [36, 112].

In addition to biological barriers, the structural stability of editing components also plays a key role in a successful edit. The 3' extension of pegRNA is very easily degraded by exonucleases, which truncates the template required for reverse transcription. To limit this damage, researchers designed engineered pegRNAs (epegRNAs) containing structured RNA motifs, such as tevopreQ. 1 or mpknot. These motifs protect the 3' extension and increase the efficiency by 3‐ to 4‐fold [293, 294, 295]. Another protein‐engineering approach is the PE7 system. It directly fuses the RNA‐binding domain of the La protein (SSB) to the editor, which helps to stabilize the 3' poly‐U tract of the pegRNA [296, 297].

### Epigenome writing and transcription control

Moving from genome editing to genome regulation is a key step in precision engineering. By separating DNA binding from nuclease activity, researchers found ways to write functional genomic states. These tools work to alter transcription and shift chromatin structure, and they can also reorganize the 3D genome architecture. All of this happens without making permanent changes to the DNA sequence. This approach helps explore the factors that control gene expression. Also, it might provide a basis for new treatments that adjust gene levels but leave the underlying DNA intact [298, 299].

Currently, most genome writing relies on catalytically inactive Cas variants, like dCas9 or dCas12. These proteins work as programmable scaffolds to bind DNA [9, 48, 300]. In the beginning, early versions of CRISPR interference (CRISPRi) just used dCas9 to physically block RNA polymerase. But this method showed quite limited efficiency in mammalian cells. To solve this problem, researchers linked dCas9 proteins to effector domains, like the Krüppel‐associated box (KRAB). The KRAB domain pulls in natural chromatin modifiers, such as KRAB‐associated protein 1 (KAP1) to build heterochromatin, and this change leads to much better gene silencing efficiency and specificity [120, 301, 302].

On the activation side, CRISPR activation (CRISPRa) has moved past simple dCas9‐VP64 fusions. Instead, researchers use more complex designs that mimic natural transcriptional recruitment. For example, systems, such as VP64‐p65‐Rta (VPR), the synergistic activation mediator (SAM), and SunTag depend on peptide arrays or modified RNA scaffolds to recruit multiple copies of transcript ional effectors. This setup plays an important role in activating genes that are otherwise hard to turn on, and it may even allow for cellular reprogramming [303, 304, 305, 306, 307] (Figure 5A). Besides, researchers developed orthogonal scaffold RNAs and Csy4‐based processing systems to manage multiplex regulation. These systems let the cell activate and repress different genes at the same time. We expect that this ability will serve as an important building block to construct complex synthetic gene circuits [308, 309, 310, 311].

**Figure 5:**
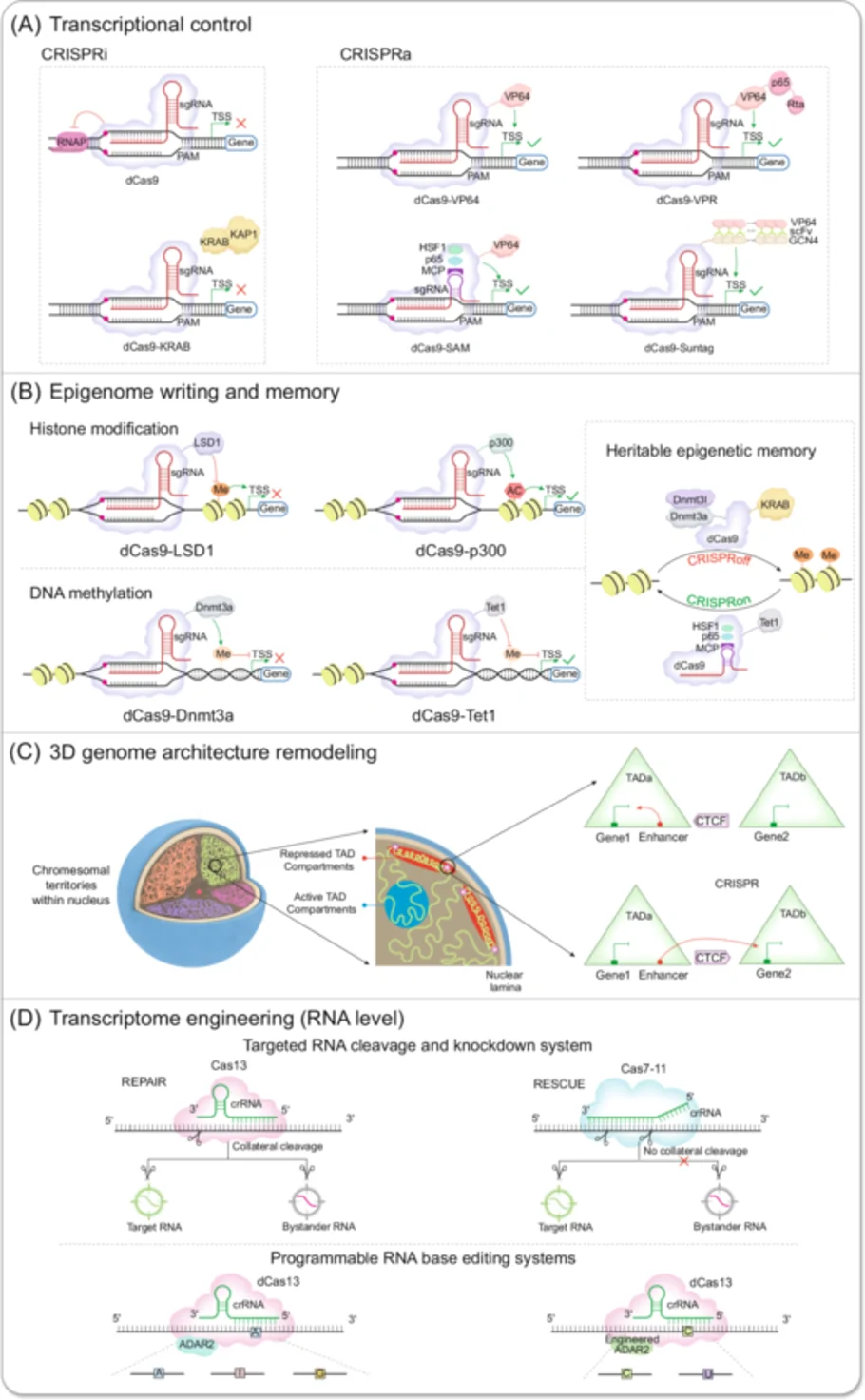
Mechanisms of precision epigenome and transcriptome engineering. (A) Transcriptional control. Catalytically inactive Cas9 (dCas9) mediates CRISPR interference (CRISPRi) either via steric hindrance of RNA polymerase or by recruiting chromatin modifiers, such as KAP1, through the Krüppel‐associated box (KRAB) domain [48]. CRISPR activation (CRISPRa) stimulates transcription utilizing either direct effector fusions (VP64, VPR (VP64‐p65‐Rta)) or multivalent recruitment architectures (synergistic activation mediator (SAM), SunTag) to synergistically assemble transcriptional machinery [304]. (B) Epigenome modulation and memory. Targeted effectors dynamically remodel chromatin without altering underlying DNA. dCas9‐LSD1 and dCas9‐p300 catalyze locus‐specific histone demethylation and acetylation, respectively. Similarly, dCas9‐Dnmt3a deposits repressive DNA methylation (Me) to block transcription, whereas dCas9‐Tet1 facilitates targeted demethylation to activate transcription. The CRISPRoff complex establishes a self‐propagating, heritable heterochromatic state via DNA methylation, which is specifically reversible by the CRISPRon system, providing long‐term epigenetic memory [47]. (C) 3D genome architecture remodeling. CRISPR‐mediated inversion of CTCF‐binding sites structurally reconfigures chromatin looping, thereby rewiring topologically associating domains (TADs) and redirecting spatial enhancer–promoter interactions [122]. (D) Transcriptome engineering. Target recognition by VI‐type Cas13 induces nonspecific collateral RNA cleavage, while III‐E type Cas7‐11 executes precise, non‐collateral RNA cleavage devoid of bystander activity [299]. Furthermore, catalytically inactive Cas13 fused to adenosine deaminases acting on RNA (ADAR) variants enables programmable RNA base editing, facilitating targeted A‐to‐I and C‐to‐U transcriptomic conversions without genomic risk [298].

In addition to generic activation or repression, epigenome writing involves the addition of enzyme‐binding sites or the removal of specific chromatin marks to manipulate regulatory logic. This approach has helped clarify the functional hierarchy of epigenetic modifications. For example, base editing of histone genes revealed that H3K27 acetylation is dispensable for the activation of differentiation‐associated genes, challenging established models [312, 313]. Tools, such as dCas9‐p300 and dCas9‐LSD1, enable programmable installation of H3K27ac or removal of H3K4me2, respectively, thereby facilitating gene activation from distal enhancers or silencing of specific regulatory elements [46, 222, 314]. Furthermore, methylation editing using dCas9‐Tet1 (demethylase) and dCas9‐Dnmt3a (methyltransferase) fusions has demonstrated therapeutic potential by reversing pathogenic epigenetic states, such as fragile X messenger ribonucleoprotein 1 (*FMR1*) gene reactivation in fragile X (FX) neurons through targeted demethylation of CGG repeats [315, 316, 317] (Figure 5B).

One major limitation of early epigenetic tools is that their effects are lost once the effector is removed. The development of CRISPRoff provided a way to establish heritable epigenetic memory. By fusing dCas9 to the KRAB and Dnmt3a/3 L domains, this system drives a heterochromatic state that can propagate itself. This state involves H3K9me3 and DNA methylation, and it remains stable for hundreds of cell divisions even after the editor is expressed for only a short time [47, 119, 217]. However, this stability largely depends on the cellular context and is often transient in vivo. For instance, in naïve pluripotent states, factors like DPPA2 act as an intergenerational safeguard to erase this induced epigenetic memory. Because of this, maintaining synthetic epialleles long‐term in complex tissues usually needs specific developmental cues or a competitive selective advantage (such as increased cell survival or proliferation). These factors help the cells overcome endogenous reprogramming barriers [318]. Although long‐term repression is possible under certain conditions, this epigenetic state is not permanent and can still be reversed. Using CRISPRon or specific anti‐CRISPR proteins allows researchers to control gene expression states over time [73, 134, 319] (Figure 5B). Beyond targeted chemical modifications, epigenome writing also involves the 3D architecture of the genome. For example, researchers have used CRISPR to invert CCCTC‐binding factor (CTCF)‐binding sites. This alters topologically associated domains and changes the connections between enhancers and promoters, which ultimately redirects gene expression through structural engineering [121, 122, 174] (Figure 5C).

In addition to DNA regulation, transcriptome engineering provides a way to achieve transient and reversible changes at the RNA level. Type VI CRISPR systems, like Cas13, are effective tools for RNA knockdown. However, they suffer from collateral cleavage activity. Once they recognize their target, they tend to degrade bystander RNAs indiscriminately, and this leads to toxicity in mammalian cells [299, 320, 321]. To address this problem, the Type III‐E effector Cas7‐11 was developed. It is a single‐protein enzyme that can cut RNA precisely without showing collateral activity [299, 322]. Also, combining a catalytically inactive Cas13 with adenosine deaminases acting on RNA (ADAR) makes programmable RNA base editing possible, as seen in the programmable RNA editing systems REPAIR and RESCU. This approach allows for the correction of point mutations at the transcript level and avoids genomic risk [323, 324] (Figure 5D). Recently, the discovery of ultracompact variants like Cas13bt has helped to fit these high‐fidelity RNA editors into a single viral vector. This step makes them much more practical for precision engineering in vivo [325, 326].

### Organellar genome engineering

The engineering of organellar genomes, particularly mitochondrial DNA (mtDNA), is challenging due to several biological barriers, necessitating strategies that are fundamentally distinct from nuclear genome editing. Unlike the nucleus, which readily supports the import RNA‐guided nucleases, such as Cas9 and repair‐mediated editing, mammalian mitochondria lack efficient HDR machinery. Consequently, DSBs induced by earlier generations of mitochondrial‐targeted nucleases (e.g., mitoTALENs and ZFNs) result in rapid mtDNA degradation rather than precise sequence correction. Consequently, mitochondrial engineering has been limited to eliminating pathogenic haplotypes by shifting to heteroplasmy levels rather than precisely correcting mutations [327]. Therefore, to achieve true genome writing in organelles, DSB‐based mechanisms should be replaced with novel, protein‐based architectures capable of operating within the unique constraints of the mitochondrial matrix.

During organellar editing, standard CRISPR‐Cas complexes, including bulky ribonucleoprotein complexes, cannot enter mitochondria because of their double membrane and selective protein import machinery, posing a major challenge. Studies on nucleus‐like compartments in bacteriophages have provided insights into this mechanism by demonstrating that proteinaceous shells analogous to organellar membranes render DNA pan‐resistant to RNA‐guided nucleases by physically blocking their entry [322, 328]. Therefore, protein‐based targeting should be used instead of RNA‐guided recognition to overcome this compartmentalization. By fusing functional payloads to internal trafficking proteins or appending mitochondrial targeting signals to transcription activator‐like effector (TALE) arrays, the import machinery [329] of the organelle can be co‐opted to deliver engineered effectors. This finding suggests that successful mitochondrial editors must be protein‐only systems, requiring the discovery of deaminase domains capable of functioning independently of the Cas9 R‐loop [330, 331].

Researchers achieved precise cytosine base editing in mitochondria following the discovery and engineering of the interbacterial toxin DddA (*Burkholderia cenocepacia*). The APOBEC/AID family enzymes used in nuclear BEs require ssDNA substrates. However, DddA is different, which shows cytidine deamination activity on dsDNA. To limit the harmful effects of this toxin, DddA‐derived cytosine BEs (DdCBEs) use a split‐protein architecture. In this structure, the toxic domain is divided into inactive halves, and then they are fused to TALE arrays. The halves show functional reconstitution only when two TALE units bind adjacent target sites (spaced 14–18 bp apart), which can ensure high specificity. For example, we can use a UGI to prevent uracil excision. Then, DdCBEs can lead to efficient C–G to T–A conversions. In this process, almost all product purities exceed 99.5%, while indel frequencies remain extremely low (<0.1%). This method, therefore, avoids the damage associated with nuclease‐mediated methods [330, 332, 333, 334, 335].

Researchers also developed TALE‐linked deaminases to influence A‐to‐G conversions in mitochondrial genomes. They used the evolved deoxyadenosine deaminase TadA8e in this system. However, there is a critical safety limitation in the early applications of TadA8e in the mitochondrial matrix. The enzyme has a high intrinsic affinity for RNA, which leads to widespread off‐target editing (A‐to‐I conversions) across the transcriptome. This problem showed harmful cytotoxicity and embryonic lethality in murine models. To address this problem, structure‐guided evolution was used to separate the DNA deaminase activity from RNA‐binding affinity. Engineered variants, such as split TALE‐linked deaminase (sTALED)‐V28R and sTALED‐R111S reduced RNA off‐target editing by over 99% while maintaining robust on‐target mtDNA editing. These variants narrowed the editing window to decrease bystander edits, and they play an important role in the precise modeling of diseases, such as Leigh syndrome, with high efficiency and viability [336, 337, 338]. In conclusion, the field of organellar genome engineering is expected to transition from gene editing toward precise writing. The mechanisms depend on protein‐only delivery strategies and rationally engineering of enzyme kinetics to match the unique biochemical environment of the mitochondrion [339, 340].

### Section summary

The precision era represents advanced applications of chimeric effector proteins that enable direct, site‐specific chemical modification and local sequence synthesis within the genome, rather than relying on stochastic cellular repair. However, several challenges remain. These include intrinsic catalytic promiscuity, which manifests as bystander mutations and transcriptome‐wide RNA off‐target editing; pervasive antagonism from endogenous mismatch repair (MMR) pathways; and severe delivery constraints caused by the large macromolecular size of these systems. Overcoming these limitations will require the synergistic integration of generative AI‐driven *de novo* protein design with highly specific peptide binders capable of transiently evading host surveillance machinery without inducing global mutational risks. As the fidelity of single‐base and short‐sequence editing approaches optimal specificity, the primary objective of genome engineering has shifted toward overcoming payload capacity constraints, thereby paving the way for the large‐scale integration and synthetic design mechanisms detailed in the following chapter.

## 5 GENOME WRITING: LARGE‐SCALE INTEGRATION AND SYNTHETIC DESIGN

### Overview

Large‐scale genome writing represents an advanced stage of targeted genetic manipulation, transforming the genome from a collection of isolated loci into a dynamic, programmable substrate for complex, multikilobase synthetic design. This expanded capability is powered by a diverse set of mobile genetic elements and integrase machineries, including CAST systems, OMEGA systems, recombinase‐based hybrid systems (PASTE), and recently discovered dual‐recognition bRNAs. As base and prime editors have strict insertion‐size limitations, the installation of entire metabolic pathways or therapeutic gene cassettes remains highly restricted, creating a critical biological bottleneck. As a result, the development of high‐capacity, DSB‐free integration engines has become essential. In this section, we review the mechanisms of RNA‐guided transposition and recombinase‐mediated large‐scale genome writing, outline the principles of programmable recombination driven by bRNAs, and discuss emerging applications of these tools in genomic recording and temporal biological logging.

### RNA‐guided transposition: CASTs and OMEGA

A major requirement for genome writing is the integration of multikilobase genetic payloads without relying on the stochastic DNA repair machinery of the host cell. Although base and prime editors offer high precision, their utility is kinetically constrained by limitations in insertional capacity [341]. To overcome this limitation, researchers have used mobile genetic elements that have evolutionarily co‐opted CRISPR‐Cas components to facilitate their propagation. This section analyzes the mechanisms of CASTs, which use nuclease‐deficient Cas effectors for programmable insertion, and their evolutionary ancestors, the OMEGA systems, which elucidate the structural association between transposons and adaptive immunity.

Programmable genome engineering originated with the discovery of the IS200/IS605 transposon family. Phylogenetic and structural analyses have shown that the RNA‐guided nucleases Cas9 and Cas12 evolved from the transposon‐encoded proteins IscB and TnpB, respectively [79, 342, 343, 344]. These OMEGA systems represent the minimal functional core of RNA‐guided endonucleases. TnpB, a hypercompact protein of approximately 400 amino acids, forms a ribonucleoprotein complex with a noncoding *ω*RNA derived from the right end of the transposon. This *ω*RNA contains a pseudoknot structure that functionally replaces the REC lobe found in larger Cas effectors, guiding TnpB to recognize a transposon‐associated motif and cleave the DNA [79, 345, 346, 347]. Unlike CAST systems, OMEGA systems function as nucleases that drive an excision‐dependent replication mechanism. Following transposase‐mediated excision of the element, TnpB cleaves the donor site, initiating homologous recombination that restores the transposon back into the original locus [348, 349, 350] (Figure 6A). In eukaryotes, this system is represented by the Fanzor (Fz) proteins, which have been engineered alongside TnpB variants using generative AI models, such as Evo to create hypercompact editors for mammalian genome editing [11, 50, 54, 351].

**Figure 6:**
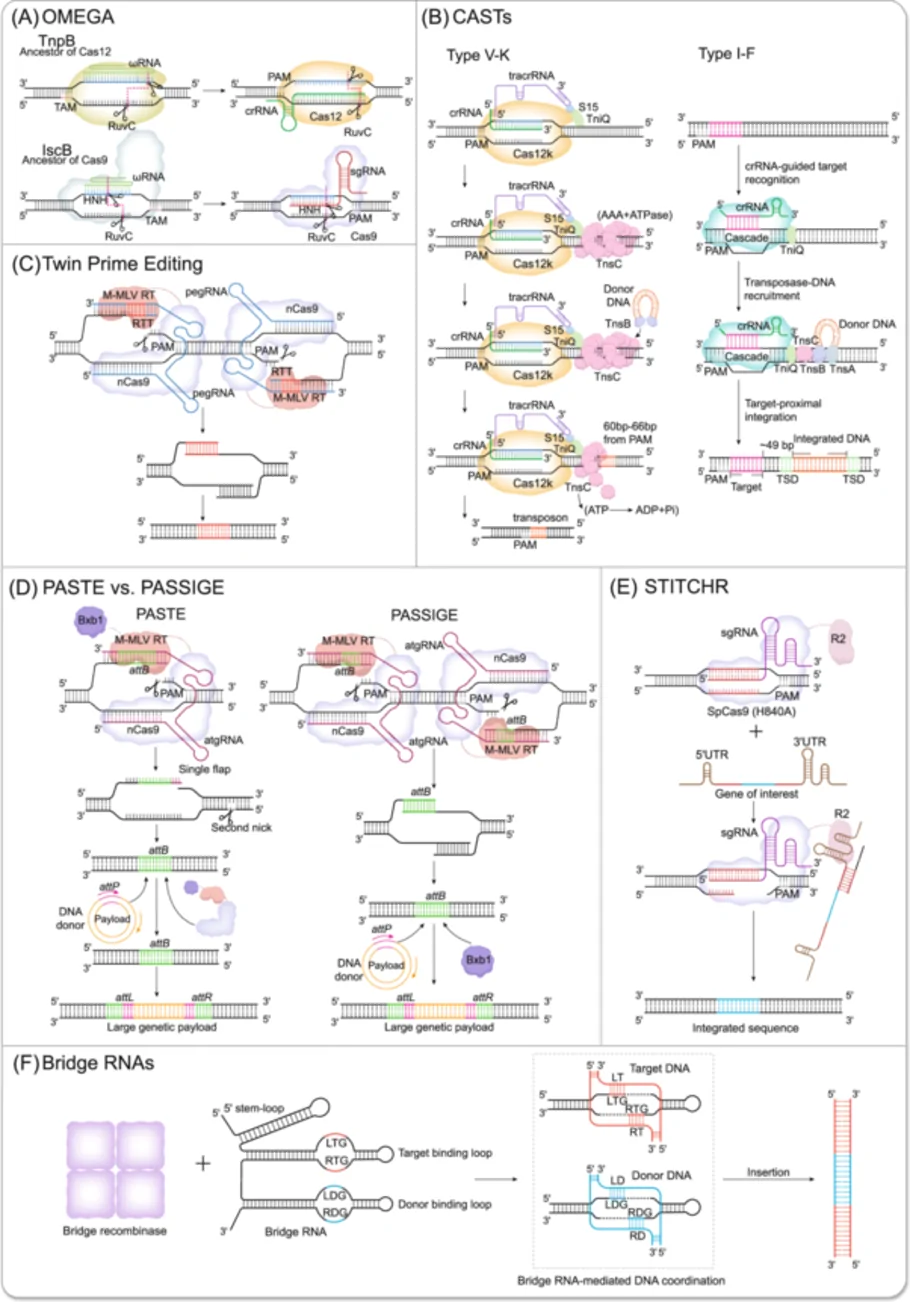
Mechanisms of large‐scale genome writing and synthetic design. (A) Obligate mobile element‐guided activity (OMEGA). TnpB and IscB are the evolutionary ancestors of Cas12 and Cas9. They work together with *ω*RNAs to find transposon‐associated motifs (TAMs) and lead to targeted DNA cleavage [79]. (B) CRISPR‐associated transposases (CASTs). In Type V‐K systems, the nuclease‐deficient Cas12k and TniQ initiate the formation of TnsC helical filaments, which then recruit TnsB to integrate the payload 60–66 bp after protospacer adjacent motif (PAM). In Type I‐F systems, a Cascade complex comprising several subunits is used to direct TnsA, TnsB, and TnsC. This results in a precise integration approximately 49 bp downstream and also creates target‐site duplications (TSDs) [38]. In *V. cholerae* Tn6677, TniQ attaches to the Cascade R‐loop, which eventually pulls in TnsC, TnsA, and TnsB for the integration process. (C) Twin prime editing. Two prime editing guide RNAs (pegRNAs) are used to lead Cas9 nickase–reverse transcriptase modules to generate complementary 3' DNA flaps, and this helps set up the site‐specific attachment loci [341]. (D) Programmable addition via site‐specific targeting elements (PASTE) and prime editing‐assisted site‐specific integrase gene editing (PASSIGE). In the PASTE method, an nCas9‐reverse transcriptase module is physically joined with a Bxb1 integrase. This links the making of the local attB site generation with attP payload integration. PASSIGE, however, separates these two steps using a Bxb1 recombinase that is not fused to the module. Both of these systems result in attL and attR junctions on the sides [116]. (E) Scarless targeted integration utilizing target‐primed reverse transcription (STITCHR). A fusion of SpCas9(H840A) and the R2 retrotransposon performs target‐primed reverse transcription, which allows for payload integration without leaving a scar. (F) Bridge RNAs. The IS110 recombinase functions with a bispecific bridge RNA containing two independent parts: target‐binding loops (left target guide/right target guide (LTG/RTG)) and donor‐binding loops (LDG/RDG). This setup allows the system to recognize two sites simultaneously, which lines up the target and donor DNA molecules for direct recombination without needing double‐strand breaks (DSBs) [40].

Unlike the nuclease‐dependent mechanism of OMEGA, CAST systems involve Tn7‐like transposons that hijack CRISPR‐Cas systems solely for target recruitment, repurposing the Cas effector from an immune nuclease into a DNA‐binding module [38]. Type V‐K systems, such as ShCAST (*Scytonema hofmannii*), use a nuclease‐deficient Cas12k effector. Upon target recognition, the protein TniQ associates with the Cas12k effector complex and acts as a recruitment hub for the AAA+ ATPase TnsC [78, 352]. Nucleated by TniQ, TnsC polymerizes into helical filaments along the DNA to remodel the duplex. It is this TnsC filament that subsequently recruits the transposase TnsB, driving the integration of the payload 60–66 bp downstream of the PAM site [337, 353, 354, 355]. However, Type V‐K systems employ a copy‐and‐paste mechanism that frequently yields cointegration products containing plasmid backbone sequences, resulting in significant off‐target integration in heterologous hosts [38, 356, 357, 358, 359, 360, 361].

Type I‐F CASTs, such as VchINT (*Vibrio cholerae*), offer a precise cut‐and‐paste mechanism. These systems employ a multi‐subunit CRISPR‐associated complex for antiviral defense (Cascade) complex (Cas8–Cas7–Cas6), in which TniQ is integrated into the complex, enabling direct recruitment of the transposition machinery without the need for extensive filament formation [37, 362]. Consequently, payloads up to 10 kb can be inserted approximately 48–50 bp downstream of the target site with over 99% on‐target specificity [356, 363] (Figure 6B). The recruitment process is also highly regulated. For instance, in Type I‐B systems, R‐loop formation triggers a conformational change in Cas6 that exposes the TniQ‐binding site, effectively licensing transposition only after successful target engagement [364, 365, 366].

Beyond these canonical systems, RNA‐guided integration machineries have expanded to include novel scaffolds for synthetic genome design. High‐resolution terascale clustering has identified CasMu systems, which associate CRISPR effectors with Mu transposases, and TIGR systems, a novel family of RNA‐guided DNA‐targeting nucleases derived from IS110 transposases that employ unique dual‐spacer tigRNA architectures [159, 367]. Although bacterial CASTs were initially inefficient in mammalian cells, directed evolution has yielded variants, such as evoCAST with substantially improved activity in human cell lines [368, 369]. Furthermore, these systems have been shown to perform writing functions in vivo via the MetaEdit platform, which successfully integrated metabolic pathways into the genomes of complex microbiome species [370, 371, 372]. Collectively, these technologies represent a shift from the destructive cut of nucleases to the constructive paste of integrases, thereby addressing the insertional payload limitations inherent to conventional genome engineering approaches [373, 374].

### Recombinase‐mediated large‐scale writing

Although RNA‐guided transposition systems are effective for integration, most mammalian genome‐writing methods use hybrid strategies that combine the programmable specificity of CRISPR with the high catalytic power of site‐specific recombinases. This approach addresses the efficiency–precision–payload trade‐off that constrains nuclease‐based methods, in which strategies, such as HDR and HITI, are limited by cytotoxicity, limited cargo capacity, and cell‐cycle dependence [96, 251, 375]. To overcome these barriers, a two‐step, site‐specific attachment locus strategy has been implemented, decoupling the search function of the editor from the write function of the integrase, enabling deterministic, multikilobase insertions [39].

The development of twin prime editing (twinPE), which employs two pegRNAs to synthesize complementary DNA flaps, established the foundational proof of concept. Using this mechanism, recombinase attachment sites (e.g., attB) can be precisely installed with >80% efficiency, creating a docking site for serine integrases such as Bxb1 to mediate payload insertion or genomic inversion [213, 341, 376] (Figure 6C). To translate this approach into a therapeutic modality, the PASTE system directly fuses the Cas9 nickase–reverse transcriptase module to the integrase. This strategy enables the integration of payloads ranging from approximately 1 to 36 kb with 50%–60% efficiency in dividing cells and substantial activity in nondividing primary hepatocytes, effectively overcoming the cargo capacity limitations of therapeutic genome engineering [39, 377, 378]. However, the fusion of bulky effector domains introduces delivery challenges and steric constraints. Subsequent optimization has resulted in prime editing‐assisted site‐specific integrase gene editing, which separates the writing steps and uses evolved and engineered recombinases (eeBxb1). Through PACE, eeBxb1 was optimized for mammalian activity, achieving integration efficiencies for payloads up to 10.5 kb that were approximately 16‐fold higher than those of fusion systems [116, 273] (Figure 6D). The success of these systems is determined by the processivity of the initial PE step. Advanced editors, such as PE6d, have been developed to write structured loxP sites with 23‐fold higher efficiency than standard editors, enabling robust in vivo writing using dual‐AAV delivery [36, 379].

Distinct from the site‐specific attachment locus strategy of serine integrases, novel systems based on retrotransposons and eukaryotic transposases provide alternative mechanisms for large‐scale genome writing, often with unique advantages, such as scarless insertion. The gene editing platform STITCHR reprograms the target‐primed reverse transcription mechanism of R2 retrotransposons. By fusing the R2 protein to SpCas9(H840A), STITCHR enables scarless integration of payloads up to 12.7 kb without leaving residual *att* sites, a limitation of recombinase‐based methods. Notably, this mechanism is independent of cell cycle, retaining efficacy in contexts where HDR is ineffective [82, 150, 380] (Figure 6E). In plant genome engineering, the TATSI system integrates Cas nucleases with the rice Pong transposase to execute a cut‐and‐paste mechanism. In this system, Cas9 generates a target DSB that captures the excised transposon [381], achieving targeted insertion efficiencies of 35.5% in *Arabidopsis* and substantially outperforming conventional knock‐in methods in crops [381, 382, 383, 384].

In addition to therapeutic applications, recombinase‐mediated writing is crucial for bioprospecting and metabolic engineering, which capture and transfer large biosynthetic gene clusters (BGCs) [385]. Recently developed workflows combine in vivo excision with integrase‐mediated writing to clone BGCs exceeding 80 kb. For example, the Cas9–BD system enables the excision of large genomic clusters, such as the 81.2 kb FK506 cluster, which can subsequently be captured and integrated into heterologous hosts using the *φ*C31 integrase [133, 386]. For the direct assembly of large operons without the need for replication intermediates, linear–linear homologous recombination mediated by full‐length RecE/RecT systems outperforms Red *αβ* recombineering [387, 388, 389]. Using this approach, a 26‐kb magnetosome pathway was transferred into *Rhodospirillum rubrum*, thereby conferring the ability to biomineralize magnetic nanostructures [390, 391, 392].

Finally, the genome can be transformed into a dynamic recording device and a fluid substrate for evolutionary engineering using recombinases. The Polylox system uses a synthetic cassette of alternating loxP sites and barcodes. Stochastic recombination generates over 1.8 million unique codes upon Cre induction, effectively writing lineage information into the genome that can be read through long‐read sequencing [393, 394]. In the context of synthetic genomics, the Sc2.0 project uses loxPsym sites embedded throughout synthetic yeast chromosomes to enable Synthetic Chromosome Rearrangement and Modification by LoxP‐mediated Evolution (SCRaMbLE). This inducible system drives large‐scale deletions, inversions, and translocations, enabling the rapid evolution of complex traits [55, 395]. Similarly, PE has been used to install loxPsym sites into repetitive LINE‐1 elements in mammalian cells, enabling the randomization of genomic architecture to map gene essentiality [56, 396]. Collectively, these recombinase‐mediated strategies represent a transition from conventional gene editing to advanced techniques that enable the genome to be written, recorded, and restructured at scale.

### Programmable recombination via bRNAs

Recently, the discovery of RNA‐guided systems derived from the IS110 family of insertion sequences brought new changes to genome writing. This system is fundamentally different from CRISPR‐Cas nucleases and CASTs. Although CASTs and hybrid integrase systems, such as PASTE, enable large‐sequence insertion, they usually need complex multiprotein effector complexes or multistep site‐specific attachment locus strategies. In contrast, the IS110 system requires only a single recombinase enzyme and a bispecific noncoding RNA to change DNA sequences. We consider this bRNA‐guided mechanism as a third generation of RNA‐guided technologies. It provides a simple and unified platform for large‐scale insertion, excision, and inversion. It does not need DSBs or host‐specific repair factors [40, 41, 397].

The IS110 system contains a bRNA whose structure is fundamentally different from the sgRNA of Cas9. The bRNA has two separate programmable internal loops. One is a target‐binding loop that base‐pairs with the target genomic site, and the other is a donor‐binding loop that base‐pairs with the DNA payload before insertion. Previous studies showed that the IS110 recombinase, such as IS621, binds to bRNA with high affinity (dissociation constant KD ≈ 2.1 nM). They form a ribonucleoprotein complex that recognizes both the target and donor DNA simultaneously [40]. As shown in Figure 6F, this dual‐recognition mechanism allows the system to directly bridge two dsDNA molecules. In contrast, CRISPR‐based editing uses guide RNAs that only find the genomic target. For the donor template, it has to rely on passive cellular diffusion or physical tethering for incorporation.

Based on structural and phylogenetic analyses, the IS110 system was recently found to be evolutionarily related to TIGR‐Tas defense systems and Box C/D small nucleolar RNAs (snoRNAs). The RNA‐binding domain of the IS110 recombinase shares strong homology with the nucleolar protein (Nop) domain family found in TIGR systems. Both types use a split/tandem spacer structure anchored by conserved Box C and Box D motifs. However, they work in different ways. TIGR systems use this structure to bind opposite strands of a single DNA target for defensive cleavage, whereas IS110 systems use the Nop‐domain scaffold to bind two separate DNA molecules for recombination [367, 398]. This suggests that the Nop‐domain architecture may serve as a foundational platform to design different DNA manipulation tools beyond simple nuclease activity [367].

Functionally, the IS110 system operates through a cut‐and‐paste mechanism involving a circular DNA intermediate. This makes different genomic writing modes possible. Our field recently found that this system can insert approximately 5 kb (4.85 kb) of genetic payloads by changing the bRNA loops. This helps solve the cargo‐capacity limits of current viral vectors. In addition to insertion, the system can also be programmed to cut out DNA segments or flip genomic orientations. The efficiency is higher than 90% in bacterial models [40, 399]. Nanopore sequencing has demonstrated very high specificity. Over 96% of insertions happen right at the programmed target site. Specificity can be further improved by extending the base‐pairing length of the RTG loop. This stops off‐target binding because it increases the thermodynamic threshold for recognition [40, 148].

The IS110 system addresses a few big problems of previous genome‐engineering methods. Structurally, the enzyme functions as a homodimer and is substantially smaller than Cas9 or multi‐subunit CAST complexes, making delivery into compact vectors, such as AAV, more feasible [41, 103]. Biologically, the system is autonomous and does not need host‐specific factors. This is a clear difference from HDR‐dependent methods that only work in dividing cells. Right now, this system is mostly tested in bacterial and cell‐free systems. However, the bRNA mechanism gives us a direct and non‐genotoxic strategy for programmable recombination. We hope that it will lead to safer and large‐scale genome writing in the future [41].

### Genomic recording and temporal logging

Since the advent of genome writing, the genome has transitioned from being merely a substrate for modification to a dynamic, writable medium for information storage. This capability, termed genomic recording, allows cells to autonomously log their lineage history, transcriptional states, and environmental exposures into their own DNA sequences [400]. These technologies offer precise, sequential digital recording solutions that fundamentally alter the ability of the cell to reconstruct its biological history.

Genomic recording is based on the natural CRISPR‐Cas adaptive immune system, which functions as a molecular memory device. Bacteria and archaea chronologically record viral infection histories by integrating foreign DNA fragments, or spacers, into CRISPR arrays in a polarized manner [5, 337, 401]. This natural mechanism, mediated by the Cas1–Cas2 integrase complex, provided the blueprint for a synthetic digital storage system. By overexpressing Cas1–Cas2, researchers demonstrated in vivo digital data storage, encoding a digital movie into the *E. coli* genome with high reconstruction accuracy, thereby validating that the physical order of spacers effectively encodes temporal data [358, 402] (Figure 7A). The Cas9 effector regulates the specificity of this adaptation process, ensuring that only PAM‐compliant targets are recorded [404]. Advanced iterations, such as the Retro‐Cascorder, integrate this spacer‐acquisition logic with retron‐mediated reverse transcription. In this system, transcriptional events generate DNA receipts from RNA barcodes, which are subsequently integrated into the array, successfully reconstructing the temporal order of gene‐expression events [313, 405].

**Figure 7:**
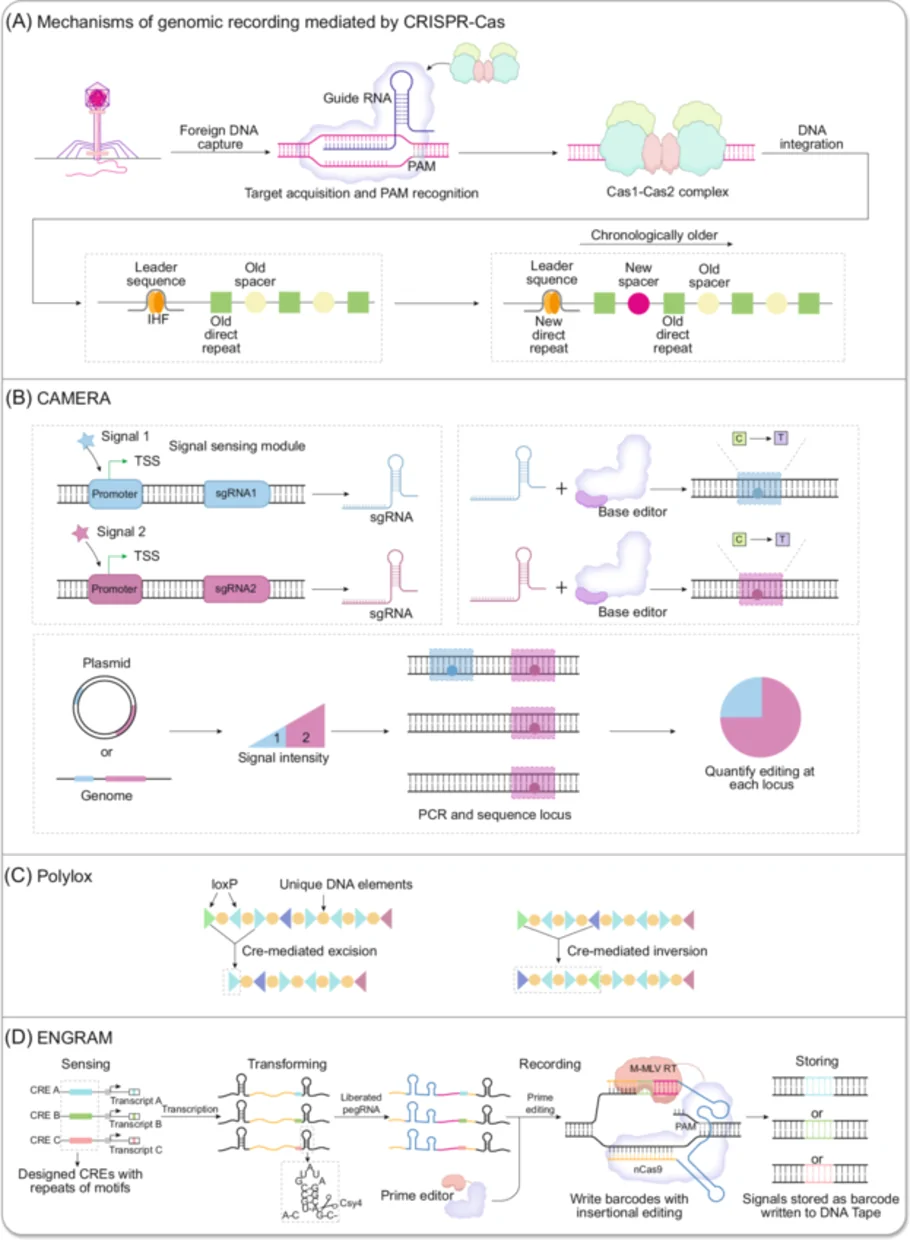
Mechanisms of precision genomic recording and temporal logging. (A) Clustered regularly interspaced short palindromic repeats‐CRISPR‐associated (CRISPR‐Cas)‐mediated genomic recording. After target acquisition and the recognition of a protospacer adjacent motif (PAM), the Cas1‐Cas2 integrase complex captures foreign DNA to integrate it as a novel spacer into the host CRISPR array. This polarized integration takes place specifically next to the leader sequence. As a result, the previously acquired spacers are chronologically displaced downstream, which creates a sequential temporal record [402]. (B) CRISPR‐mediated analog multistate environmental recording apparatus (CAMERA)‐mediated analog recording. In this system, different biological signals activate specific promoters to drive the transcription of corresponding sgRNAs. These single‐guide RNAs (sgRNAs) then guide base editors to catalyze targeted nucleotide conversions at specific plasmid or genomic loci. The cumulative frequency of these edits can serve as a quantitative measure to reflect the intensity and duration of the integrated signal [400]. (C) Polylox‐mediated structural barcoding. Cre recombinase catalyzes stochastic excision and inversion events across a genetic cassette, which consists of unique DNA elements separated by alternating loxP sites. These structural rearrangements produce extensive combinatorial sequence diversity to track cellular lineages [393]. (D) Enhancer‐driven genomic recording of transcriptional activity in multiplex (ENGRAM)‐mediated multiplexed recording. Here, signal‐responsive cis‐regulatory elements control the transcription of specific prime editing guide RNAs (pegRNAs) that are flanked by Csy4 hairpins. After the Csy4 endoribonuclease cleaves these hairpins, the released pegRNAs direct a prime editor (composed of Cas9 and a reverse transcriptase) to insert signal‐specific barcodes into a predefined genomic locus. Through this process, multiplexed transcriptional histories are permanently stored within the DNA [403].

In addition to integration‐based systems, a class of analog recorders has been developed to show the duration and magnitude of biological signals through the accumulation of mutations over time. For example, early systems such as mSCRIBE used self‐targeting guide RNAs (stgRNAs), and these RNAs lead Cas9 to mutate their own encoding loci. The accumulation of random indels at these loci plays a role as a quantitative record of signal intensity, which can be used to record the history of inflammation or the depth of lineage [406, 407]. To make the information capacity larger, the MARC1 system was developed. It uses homing CRISPR arrays to produce a theoretical diversity of 10^74^ heritable barcodes, which lead to the reconstruction of developmental lineage trees of the whole organism [394, 408]. However, almost all of these systems rely on DSBs, which increase genotoxic risks. To reduce this damage, the CRISPR‐mediated analog multistate environmental recording apparatus (CAMERA) systems use BEs to record signals as specific nucleotide changes (e.g., C–G to T–A). They successfully record Wnt signaling dynamics and environmental inputs and do not cut the DNA backbone [310, 400, 409] (Figure 7B). To achieve continuous recording and avoid saturation, systems such as EvolvR and helicase‐assisted continuous editing (HACE) link nickases with error‐prone polymerases or processive helicases. This plays an important role in ensuring that the nucleotides change over long genomic windows, which helps to track evolutionary depth [246, 410, 411].

Moving beyond stochastic mutagenesis, recombinase‐mediated systems enable high‐fidelity structural recording. As described previously, alternating loxP sites and barcodes in the Polylox system enable stochastic recombination upon Cre induction, generating over 1.8 million potential barcodes. Using this strategy, the lineage of hematopoietic stem cells can be traced in vivo, revealing clonal mosaicism in adult tissues [393, 395] (Figure 7C). Similarly, the iMAP system employs large arrays of guide RNAs that undergo recombination to express unique barcodes, facilitating high‐throughput functional logging of tumor evolution [412, 413]. Although these systems provide robust lineage resolution, they generally lack the capacity to store sequential, time‐resolved information.

The most advanced paradigm in genomic recording involves Typewriter systems that employ PE to write precise, ordered digital information. In the context of biological information storage, this explicitly highlights the operational distinction between editing and writing: whereas traditional editing intrinsically acts as a destructive process that erases prior mutations through progressive deletions or overwriting, true genome writing functions constructively by sequentially inserting new DNA without destroying previous historical records, enabling continuous, high‐density information accumulation [414, 415]. These systems overcome the stochastic nature of earlier tools by inserting sequential symbols into a predefined genomic tape. By coupling the insertion of a barcode with a key sequence that activates the subsequent writing site, this architecture enforces a strict 5′‐to‐3′ recording order, facilitating the reconstruction of lineage trees with Sulston‐like resolution [293, 392]. When combined with signal‐responsive enhancers in the ENGRAM system, this technology can digitize cellular signaling history. ENGRAM combines biological inputs with pegRNA expression, enabling the system to distinguish between serial, layered, and pulsed exposure patterns by reading the sequence of symbols on the DNA tape [403, 416] (Figure 7D).

Finally, to ensure the applicability of these recording systems, advanced readout technologies and epigenetic alternatives should be developed. Although sequence modification is permanent, tools such as CRISPRoff enable the writing of heritable but reversible epigenetic memory via DNA methylation and H3K9me3 deposition [47, 192]. To decode these complex histories, spatial transcriptomics methods, such as MEMOIR, employ in situ hybridization to map lineage history directly within tissue architecture [295, 417], whereas Direct Capture Perturb‐seq enables high‐throughput sequencing of guide RNAs and barcodes alongside the single‐cell transcriptome [283, 418]. Collectively, these technologies have transformed the genome from a mere template into a dynamic recording device.

### Section summary

Large‐scale genome writing represents a major engineering achievement, enabling deterministic and targeted excision or integration of large genomic sequences using highly coordinated, multi‐component effectors that function independently of host homologous recombination pathways. However, the translation of these bulky bacterial machineries into eukaryotic environments remains a major challenge. Common limitations include severe reductions in integration efficiency, structural instability at insertion sites, and increased risk of vector‐backbone cointegration. To overcome these cross‐species barriers, deep‐learning‐driven structural prediction and continuous directed evolution are expected to play an important role. These methods may enable the miniaturization of recombinases and optimization of their biochemical kinetics to better match the topological constraints of mammalian chromatin. Ultimately, large‐scale and scarless genomic integration is vital to achieve the long‐term goal of synthetic genomics: the *de novo* design and bottom‐up assembly of synthetic chromosomes. Reaching this objective will require substantial advances in computational discovery and delivery architecture, which are discussed in the following section.

## 6 ENABLING TECHNOLOGIES: DISCOVERY, DELIVERY, AND VALIDATION

### Overview

Clinical and industrial implementation of precision genome engineering depends on a robust ecosystem of enabling technologies that support the discovery of novel effectors, their safe delivery into target tissues, and high‐fidelity validation of editing DSB‐free. This infrastructure includes generative AI and structural prediction models for de novo protein design; diverse delivery vehicles such as engineered (AAVs and lipid nanoparticles (LNPs) for in vivo transport; and high‐resolution multiomic sequencing pipelines for unbiased safety assessment. As modern genome writers continue to increase in structural complexity and molecular size, traditional metagenomic mining and empirical screening approaches are reaching their operational limits. Consequently, advanced computational and materials science solutions are needed to overcome viral packaging limits and the limitations of existing validation methodologies. In this section, we review the transformative role of generative AI in computational discovery, outline the physical mechanisms and optimization of advanced delivery strategies, and discuss the validation frameworks required to ensure both precision and systemic safety.

### Generative AI and computational discovery

Recently, the *de novo* generation of synthetic editors has greatly influenced the field of genome engineering. This process depends on large metagenomic data sets, structural prediction algorithms such as AlphaFold, and generative AI models, including large language models and protein language models [419, 420]. These computational technologies help address the efficiency–precision–payload optimality trade‐off. They allow researchers to design genome editors with non‐natural properties, such as hypercompactness, expanded targeting ranges, and reduced immunogenicity [421, 422].

Generative AI is no longer limited to simple classification tasks; it now plays a major role in the *de novo* design of functional biological parts, and this relies on protein language models to learn sequence–function relationships [423]. After training on large‐scale microbial sequence atlases, these models can lead to new sequences. These sequences are quite different from natural variants, but they still retain or even improve the functionality. For example, the ProGen2‐base language model was fine‐tuned on a CRISPR‐Cas atlas containing approximately 26 terabases of data and successfully generated OpenCRISPR‐1. This Type II effector has 403 mutations compared to SpCas9. However, it still maintains its structural integrity and shows similar on‐target activity. Besides, it has almost a 95% reduction in off‐target editing at known sites [49, 51] (Figure 8A). Another example is the Evo genomic foundation model, which was trained on 300 billion nucleotides and supports a context length of 131 kilobases. It can codesign functional CRISPR‐Cas complexes (both protein and RNA) and transposable elements. The resulting EvoCas9‐1 shares only 79.9% sequence identity with natural variants. This result shows that AI has the ability to look into the protein landscape and goes beyond the limits of evolution [50, 426, 428]. Some multimodal deep‐learning frameworks, such as ProMEP, combine sequence and structural contexts to predict mutational effects, which helps to make the engineering process faster even without labeled training data. This approach played an important role in the development of TnpB‐AI‐5.6 and TadA‐AI‐15.8. Compared to their wild‐type counterparts, these two variants show improved editing efficiency and relatively lower bystander activity [54, 292, 424]. In addition, models such as ProDomino enable the one‐shot design of allosteric switches. They can find the optimal insertion sites for photosensors, and this leads to the generation of strictly inducible editors [425, 429, 430, 431].

**Figure 8:**
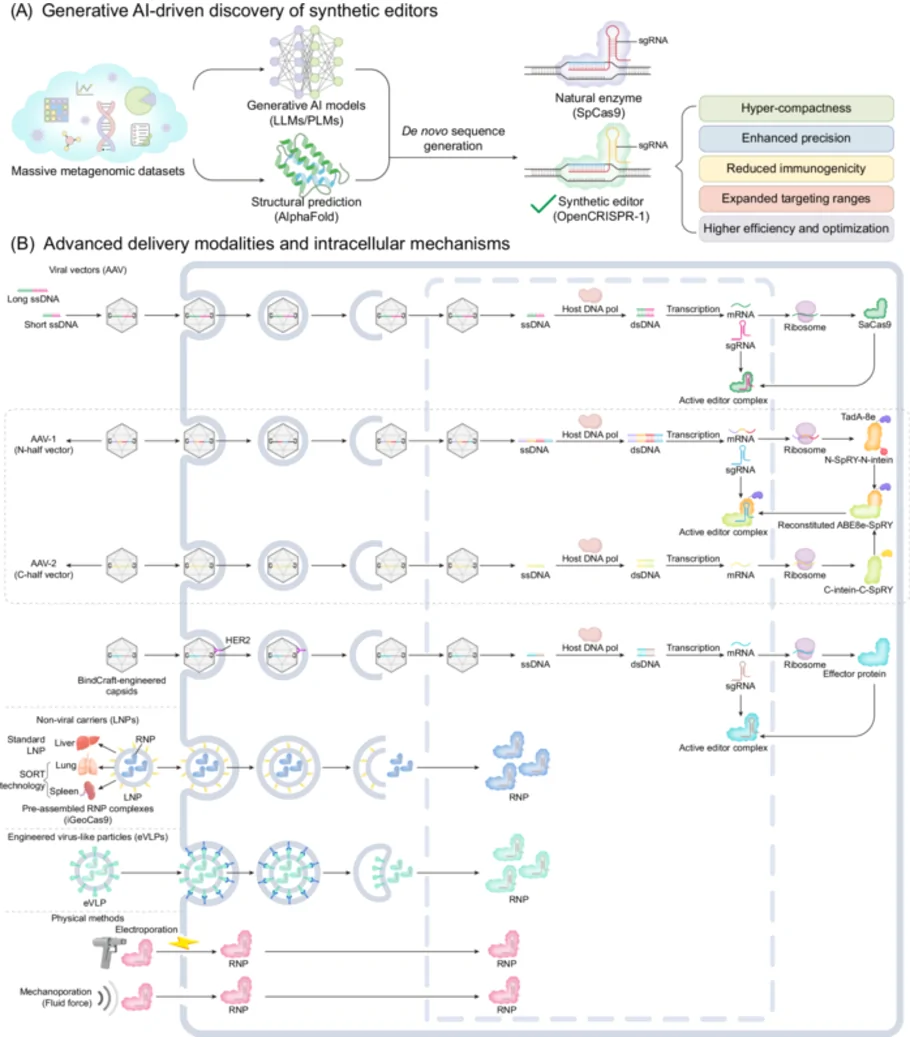
Enabling technologies for precision genome engineering. (A) Generative artificial intelligence (AI)‐driven discovery of synthetic editors. Generative artificial intelligence, integrating protein language models and structural prediction algorithms (AlphaFold), processes massive metagenomic datasets to facilitate the *de novo* sequence generation of synthetic effectors. These computationally designed editors (OpenCRISPR‐1) overcome natural enzyme (SpCas9) limitations by exhibiting optimized properties, including hypercompactness, enhanced precision, reduced immunogenicity, expanded targeting ranges, and higher efficiency [49]. (B) Advanced delivery modalities and intracellular mechanisms. To overcome strict packaging constraints, adeno‐associated virus (AAV) vectors transport short single‐stranded DNA (ssDNA) encoding miniaturized nucleases (SaCas9) for expression following host polymerase‐mediated conversion to double‐stranded DNA (dsDNA). For bulky effectors, dual‐AAV systems use split‐intein strategies to deliver editor halves, enabling post‐translational reconstitution of active base editors (ABE8e‐SpRY) via protein trans‐splicing [424]. Computationally engineered capsids (BindCraft) achieve receptor‐specific entry, exemplified by HER2 targeting [425]. Nonviral lipid nanoparticles (LNPs) utilize selective organ targeting (SORT) technology to direct biodistribution across the liver, lungs, and spleen [426]. Furthermore, LNPs, engineered virus‐like particles (eVLPs), and physical methods, comprising electroporation and fluid‐force mechanoporation, mediate direct intracellular transfer of preassembled ribonucleoprotein (RNP) complexes (iGeoCas9) to ensure transient editing activity [427].

Complementing sequence generation, the integration of AlphaFold and related structural prediction tools has transformed the rational design pipeline, enabling the engineering of enzymes without experimental crystal structures [432]. This computational discovery primarily aims to identify miniature editors compatible with AAV delivery vectors. Through computational mining of microbial data sets, NanoCas, a 425‐amino‐acid editor, was identified and packaged into a single AAV, demonstrating robust editing of the dystrophin gene in nonhuman primates [433, 434]. Similarly, AlphaFold3 was used to model the eukaryotic Fanzor2 system, guiding the truncation of RNA loops and protein engineering to increase activity by 11.1‐fold [351]. In the context of PE, AlphaFold2 predictions of RT structures enabled the engineering of compact, stabilized prime editors, whereas NUPACK algorithms established thermodynamic thresholds for predicting pegRNA efficacy [291, 379]. In addition to the editors, generative models, including RFdiffusion and BindCraft, employ *de novo* structural design to generate proteins that bind specific targets, such as the MLH1‐SB binder, which inhibits the mismatch repair pathway to boost PE efficiency by 18‐fold [125, 311, 435].

Deep‐learning models are widely applied to predict how editing events affect safety and phenotypic outcomes [436]. For example, early tools like DeepCRISPR relied on unsupervised representation learning to automate feature identification, and this approach performed much better than traditional alignment‐based methods in finding off‐target activity [170]. Later on, models such as CRISPRme began to include human genetic diversity. They revealed that population‐specific variants might create hidden off‐targets. A typical example is the high‐risk sites caused by single‐nucleotide polymorphisms that are common in certain ethnic groups [22, 245]. When looking at repair outcomes, the inDelphi model changed the old view that DNA repair is completely random. Instead, it showed that local sequence contexts can help predict these outcomes. With this capability, researchers can find gRNAs that lead to a single genotype at high frequencies, which provides a way to correct pathogenic microduplications without the need for a template [21, 168, 195, 437]. In addition, some recent computational models, such as computational analysis of phenotypic epigenomics (CAPE), use epigenomic data to predict complex phenotypic outcomes. They generally do this by pointing out the key regulatory regions that control quantitative traits [124, 263, 438, 439].

Although AI has brought huge changes to rational design, translating these computational models into actual clinical applications is still quite difficult. To achieve this goal, the field has to address several main bottlenecks. These include the lack of high‐quality training data, limited generalizability across different biological contexts, and the obvious gap between computer predictions and real wet‐lab validation.

A major problem is the shortage of high‐quality, standardized functional data. At present, deep‐learning models often depend on small and somewhat biased data sets. These data sets usually lack standardized negative samples, or they just rely on indirect proxy measures like cell survival instead of true enzymatic cleavage activity [440, 441]. Besides, because certain CRISPR systems are naturally conserved through evolution, the available sequence covariation is quite limited. This makes it hard to train models to predict non‐natural catalytic features [442, 443]. To work around this lack of data, researchers attempt to build massive, unbiased empirical data sets using ultra‐large‐scale deep sampling. For example, some studies evaluated over 740,000 gRNA‐target pairs [444, 445]. At the same time, others explore the uncharacterized sequence space found in metagenomic databases [442]. Algorithms like FLSHclust support linearithmic scaling and are able to cluster billions of proteins. This has led to the discovery of hundreds of new CRISPR‐linked modules, such as rare HNH‐nuclease variants [159, 342]. Also, by looking for biological anomalies using systematic discovery pipelines, researchers have successfully found broad‐spectrum anti‐CRISPRs [75, 446]. On the computational side, scientists try to reduce data dependency by using few‐shot active learning (like EVOLVEpro) and meta‐transfer learning frameworks (like FSFP and DeepCRISTL). These models are first pre‐trained on huge simulated or generalized data collections, and after that, they only need a small amount of highly curated empirical data to fine‐tune the system [440, 447, 448, 449, 450].

Another challenge is the poor generalizability of current models across different cell types and species. Most predictive algorithms are trained using specific in vitro settings, such as HEK293T cells. As a result, these models often fail to perform well when applied to primary tissues or in vivo environments. This issue likely occurs because the algorithms overfit to specific genomic backgrounds. They lack the capacity to account for cell‐specific factors, including differences in DNA repair abilities (such as mismatch repair dependency or DNTT expression) and varied epigenetic landscapes [440, 444, 451]. To address this problem, researchers have expected that multimodal, context‐aware network structures are required [452]. For example, some conditional generative frameworks, such as DNA‐Diffusion, directly include cell‐type labels in the generation process to create cell‐specific regulatory elements [453]. At the same time, RNA language models like CCLMoff attempt to extract condensed sequence grammars to find generic patterns that can be applied across different species [441]. A promising direction is the use of next‐generation deep‐ learning models, such as PrimeNet and DTMP‐prime. These tools directly add epigenomic features, including chromatin accessibility and DNA methylation states, into their neural networks. This approach may help the algorithms to learn the underlying biological mechanisms, which makes their predictions more reliable across different biological contexts [447, 454].

A final major hurdle is the apparent disconnect between in silico predictions and actual wet‐lab validation. Even when a model shows high computational fitness scores based on statistical similarity to natural proteins, this does not guarantee that the protein will function properly in vivo. Furthermore, these scores provide quite limited information regarding non‐natural catalytic efficiencies or cellular tolerance [435, 443, 449]. In addition, the use of artificial episomal screens and high‐copy plasmid transfections often leads to supersaturated expression levels that are not physiological. This condition frequently masks the true endogenous editing efficiencies [453, 455]. To close this gap, researchers have begun to rely on strict design‐build‐test loops. For example, several studies have introduced biomimetic changes to in vitro delivery methods, using mRNA electroporation instead of plasmid transfections to better mimic the actual behavior of the components in vivo [455, 456]. More attention is also being paid to the validation of endogenous sequences using tools like EXTRA‐seq or single‐cell MPRAs (scMPRA) to understand cellular heterogeneity [453, 457, 458]. Additionally, some newer modular AI systems combine basic protein language models with top‐layer regression networks. Because these networks are trained solely on real experimental activity data, they help link the computational designs to actual biological functions [443]. It is also observed that interpretable AI models are required to ensure that the computational predictions match real biological mechanisms [441, 450, 454, 459]. Recently, large language model (LLM)‐driven agents have even started to automate the connection between computational design and laboratory testing. Systems such as CRISPR‐GPT use multi‐agent setups to handle the entire genome‐engineering workflow, from the initial design down to the actual execution. This progress suggests that pairing AI with solid empirical feedback is expected to bridge the gap between computational predictions and actual laboratory outcomes [52].

### Advanced delivery strategies

Moving precision genome engineering from in vitro proof‐of‐concept studies to actual therapeutic applications is still limited by the balance between efficiency, precision, and payload capacity. The molecular mechanisms of editing have advanced rapidly. However, the ability to deliver these complex machineries into target cells remains a major limitation, especially for large‐scale genome writing. At present, the field is shifting from simple nuclease delivery to the transport of bulky fusion proteins and multikilobase templates. To make this work, researchers have to rely on a variety of different delivery strategies, such as viral vector engineering, nonviral synthetic carriers, and physical mechanoporation methods [35, 36, 460].

AAV vectors are still the standard method for in vivo delivery, mainly because of their low pathogenicity and broad tissue tropism [240]. But they have a strict packaging limit of about 4.7 kb, which poses a serious physical barrier [153, 461]. This limitation becomes quite obvious in genome‐writing applications. These applications usually need large effectors, like prime editors or base editors (BEs), along with guide RNAs and regulatory elements. To solve this problem, researchers have started to discover and engineer miniaturized genome‐editing effectors. For example, Staphylococcus aureus Cas9 (SaCas9, which is about 3.2 kb) proved that single‐vector delivery is possible, and this system showed efficient in vivo editing in liver and muscle tissues [242, 461, 462](Figure 8B). After further engineering, even more compact variants were developed, like evoCjCas9 (from Campylobacter jejuni). This variant can be packed into a single AAV together with base‐editing domains [463], and it results in high editing efficiencies in hepatocytes [235, 463]. Also, metagenomic mining helped extend this miniaturization trend to Type V and OMEGA systems. For instance, ultracompact variants like Cas12f (e.g., Un1Cas12f1) and Cas12j (e.g., CasPhi), as well as ancestral IscB and TnpB proteins (less than 500 amino acids), have been designed to fit inside AAVs. This leaves enough room for multiplexed guide RNAs or large donor templates, which can directly help us carry out complex gene‐correction strategies [155, 157, 345, 348, 464]. In transcriptome engineering, compact RNA effectors like Cas13bt and Cas7‐11 have solved the size problems of older Cas13 variants. These compact effectors allow researchers to perform high‐fidelity RNA editing using single‐vector systems [326, 465].

For large genome‐editing machineries that cannot be easily miniaturized without a loss of function, such as full‐length prime editors and ABEs, split‐intein strategies have become a common solution. This approach divides the large effector protein into N‐terminal and C‐terminal halves. These halves are then fused to split‐inteins, like those derived from *Nostoc punctiforme*. When delivered together via two separate AAVs, a process known as protein trans‐splicing pieces the fragments together to form the full‐length active enzyme [424, 466, 467] (Figure 8B). This dual‐vector strategy shows clear results in practice. For example, it can deliver ABEs to correct pathogenic mutations in the heart and the retina with relatively high efficiency [108, 468, 469, 470]. In the case of prime editing (PE), dual‐AAV systems also successfully fixed mutations in the central nervous system and the retina [112].

However, split‐intein strategies still face several biophysical and immunological limitations for macromolecular delivery. First of all, the efficiency of protein trans‐splicing is highly dependent on the local context and is often quite limited. The splicing process requires specific amino acid residues, such as cysteine, serine, or threonine, right at the junction sites [467, 471, 472]. Because these native extein sequences must be kept, they often leave behind amino acid scars or footprints. These extra residues can easily destabilize the rebuilt editor or lead to a drop in its catalytic activity [473, 474].

Besides, dividing large enzymes in an artificial way can disrupt their natural folding dynamics. Splitting a protein inside complex structural domains often exposes hydrophobic cores that are normally hidden. This causes the separated fragments to take on partially disordered states. These misfolded intermediate pieces tend to form inactive beta‐sheet aggregates, and these aggregates compete directly with the normal splicing pathway [467, 475, 476, 477]. In addition, the strict packaging limits of AAV vectors often restrict the choices for finding the best surface‐exposed split sites. This limitation worsens the problem of incomplete trans‐splicing, leading to stoichiometric imbalances and the buildup of unreacted, truncated half‐proteins inside the cell [471, 478, 479, 480].

There is also an important immunogenic risk, largely because inteins come from microbes. After trans‐splicing is complete, the leftover non‐mammalian intein parts and any uncleaved fragments can remain in the cell. The proteasome may process these foreign peptides and present them to CD8 + T cells via major histocompatibility complex (MHC) class I molecules. This process can trigger cytotoxic immune responses against the treated tissues [478, 479, 481, 482]. To compensate for the low splicing efficiency, researchers often need to apply extremely high doses of AAVs, which in turn leads to even more vector‐associated immunotoxicity [474].

Therefore, it is usually necessary to optimize the structure of the editor itself. For example, removing the RNaseH domain in PE6 variants helps to improve the packaging efficiency and processivity. This kind of structural change allows researchers to avoid those problematic split sites [36, 112, 294, 379]. Beyond addressing the cargo capacity, recent efforts focus on capsid engineering and generative AI to make AAVs more effective. Directed evolution has led to variants like Ark313, which show much better transduction in T cells [483]. At the same time, AI pipelines such as BindCraft make it possible to perform the *de novo* design of capsids that bind to specific receptors, like HER2. This approach helps to bypass the need for traditional high‐throughput screening [103, 260, 425] (Figure 8B).

Complementing viral vectors, lipid nanoparticles (LNPs) have emerged as modular, nonimmunogenic delivery platforms, particularly following the clinical validation of LNP‐mediated CRISPR therapeutics [484]. Unlike AAVs, LNPs enable repeat dosing, which is a key advantage for achieving cumulative therapeutic effects with BEs without eliciting neutralizing immunity [110, 485, 486]. Although standard LNPs naturally accumulate in the liver, the selective organ targeting technology has overcome this limitation by modulating the internal charge of the nanoparticle. The addition of specific cationic or anionic lipids shifts biodistribution to the lungs or spleen, respectively [426, 487] (Figure 8B). Furthermore, the incorporation of conjugation‐competent lipids enables the formation of protein coronas that target hematopoietic stem cells in the bone marrow, facilitating in vivo genome editing for hemoglobinopathies [488, 489]. The versatility of LNPs extends to the delivery of preassembled ribonucleoprotein (RNP) complexes, which provide transient activity to minimize off‐target effects, provided that thermostable Cas9 variants, such as iGeoCas9, are employed to withstand formulation processes [277, 427] (Figure 8B). Additionally, novel nanocomplex formulations exploiting active transcytosis pathways have demonstrated the feasibility of oral delivery for colorectal cancer therapies [490].

When editing primary immune cells *ex vivo*, physical and mechanical delivery methods can cause much less toxicity than viral transduction. At present, electroporation of RNP complexes is the standard way to generate engineered T cells and hematopoietic stem cells. Editing efficiency can reach over 95% after the optimization of nuclear localization signals and buffer compositions [105, 491, 492]. However, the throughput of electroporation remains limited. To address this problem, viscoelastic mechanoporation uses fluid forces to transiently open cell membranes, which helps to deliver large cargoes to hundreds of millions of cells per minute while maintaining high viability [242, 493]. In addition, hybrid approaches that combine RNP electroporation with AAV6‐mediated donor delivery have shown strong results in genome‐writing applications. For example, they can participate in targeted CAR transgene integration [235, 494]. At the same time, fully nonviral workflows using linear dsDNA templates are expected to simplify manufacturing and reduce process complexity [199, 377, 495, 496].

Researchers have also developed new vector architectures for delivering large machineries, such as CASTs and integrases for multikilobase insertions. Engineered virus‐like particles (eVLPs) have viral infection efficiency and the transient safety profile of RNPs, and this combination leads to strong base editing with almost no off‐target activity [325, 497, 498]. In the field of microbiome engineering, bacterial conjugation systems can be used to transfer CAST‐containing plasmids up to 10 kb. This method allows precise integration in complex microbial communities [356, 363, 371, 499]. Similarly, engineered phage delivery systems can influence the species‐specific editing of gut bacteria [370, 500]. In conclusion, the delivery methods may change from a universal vector approach to a diverse delivery ecosystem [75, 501, 502]. Payload miniaturization, vector optimization, and mechanical innovation play a role in making synthetic genome control possible [430, 503].

### Strategies for precision validation

The transition from stochastic gene disruption to high‐precision genome writing necessitates a parallel evolution in validation strategies. As genome‐engineering tools have evolved from simple nucleases to complex BEs, prime editors, and transposases, validation strategies have advanced from targeted locus amplification to unbiased, genome‐wide, and single‐cell multiomic analyses [504]. This evolution is critical for assessing on‐target efficiency and defining the safety limits of genomic interventions by detecting off‐target cleavage, structural genomic instability, and unintended functional consequences.

The field has moved beyond computational prediction algorithms, such as Cas‐OFFinder [505, 506], toward empirical, unbiased biochemical, and cellular screens to define the safety profile of genome editors. Highly sensitive in vitro methods [266, 507] serve as the first line of defense by identifying a superset of potential cleavage sites. Circularization for In vitro Reporting of CLeavage Effects by sequencing (CIRCLE‐seq) has emerged as a benchmark method for off‐target discovery, capable of detecting hundreds of potential sites that are often overlooked by prediction models. Complementary cell‐free approaches, including Digenome‐seq and SITE‐Seq, employ genomic DNA digestion to detect rare cleavage events, although they can overestimate off‐target risk by neglecting the influence of chromatin accessibility [69, 232, 508]. To determine which of these in vitro–identified events occur within the complex cellular context, methods, such as GUIDE‐seq, employ double‐stranded oligodeoxynucleotide capture to label DSBs in living cells [107, 164, 509]. DISCOVER‐Seq exploits the endogenous recruitment of the DNA repair factor MRE11 to DSBs for in vivo validation, particularly in therapeutic models, enabling the identification of off‐targets with single‐base resolution and high sensitivity [80, 181, 406] (Figure 9A). Furthermore, recent advancements, such as Tracking‐seq, have addressed the low input limitation, enabling off‐target detection in rare cell populations such as stem cells and showing that Cas9 and CBEs generate mutually exclusive off‐target landscapes [295, 510].

**Figure 9:**
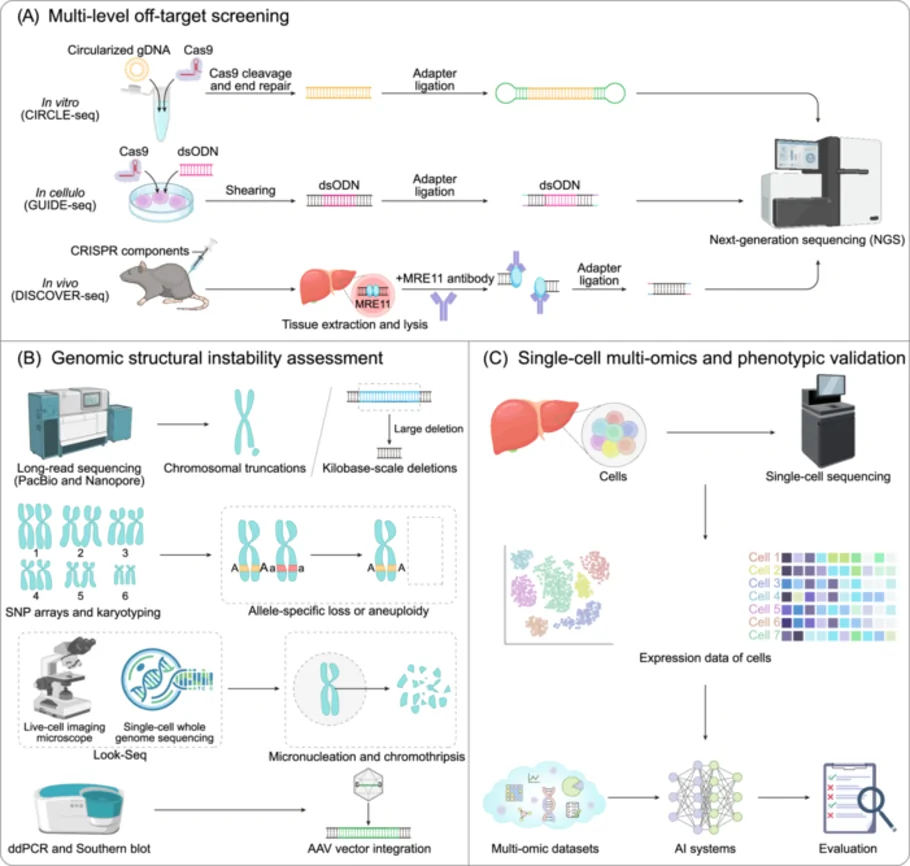
Precision validation strategies for genome engineering. (A) Multi‐level off‐target screening. Empirical workflows assess off‐target cleavage across varying biological contexts prior to next‐generation sequencing. In vitro Circularization for In vitro Reporting of CLeavage Effects by sequencing (CIRCLE‐seq) identifies potential cleavage sites utilizing circularized genomic DNA [294]. In cellulo Genome‐wide Unbiased Identification of DSBs Evaluated by sequencing (GUIDE‐seq) employs double‐stranded oligodeoxynucleotides to capture double‐strand breaks in living cells. In vivo DISCOVER‐seq detects off‐target events by tracking the endogenous recruitment of the MRE11 repair factor, followed by antibody‐mediated isolation [80]. (B) Genomic structural instability assessment. Analytical platforms resolve complex on‐target structural variants. Long‐read sequencing (PacBio, Nanopore) characterizes kilobase‐scale deletions and chromosomal truncations [25]. Single‐nucleotide polymorphism (SNP) arrays and karyotyping evaluate allele‐specific chromosome loss and aneuploidy. Integrating live‐cell imaging with single‐cell whole‐genome sequencing (Look‐Seq) identifies severe structural alterations, including micronucleation and chromothripsis [23]. Assays utilizing droplet digital PCR (ddPCR) and Southern blotting detect unintended adeno‐associated virus (AAV) vector integrations. (C) Single‐cell multi‐omics and phenotypic validation. High‐throughput single‐cell sequencing (e.g., Perturb‐seq, CROP‐seq) correlates genetic perturbations with cellular transcriptomic outputs [504]. Massive multi‐omic data sets and cellular expression profiles are computationally processed by predictive models and artificial intelligence (AI) systems (e.g., inDelphi, CRISPR‐GPT) [52]. These models autonomously evaluate complex genotype–phenotype relationships to systematically validate precise editing outcomes.

Off‐target site identification is an important step. However, conventional short‐read amplicon sequencing often has a validation gap. This is mainly because it cannot easily detect large structural variants and complex rearrangements at the on‐target locus. When using long‐read sequencing technologies like PacBio and Nanopore, Cas9‐induced breaks often show kilobase‐scale deletions and chromosomal truncations. These changes destroy PBSs, and this problem leads to false wild‐type or homozygous calls in PCR assays [194, 511]. For human embryo editing, analyses based on single‐nucleotide polymorphism (SNP) arrays and karyotyping indicate that the apparent gene correction is often just allele‐specific chromosome loss or aneuploidy [98, 205], rather than true sequence repair. Moreover, combining live‐cell imaging and single‐cell whole‐genome sequencing (Look‐Seq) shows that on‐target editing can lead to very severe problems, such as micronucleation and chromothripsis. This happens particularly in p53‐deficient cells [23, 183]. The structural validation also needs to find the capture of foreign DNA. For example, tests using droplet digital PCR (ddPCR) and Southern blotting found high frequencies of AAV vector integration at CRISPR‐induced breaks. Standard genotyping methods usually cannot detect this phenomenon [101––103] (Figure 9B). Mechanistic validation with repair‐seq showed that polymerase theta‐dependent end joining often participates in these large deletions. This finding may offer some clues to help researchers reduce genotoxicity [100, 512].

With the field moving to genome‐writing tools, the validation targets are also changing. We now need to check the purity of the products, collateral damage in the transcriptome, and integration fidelity. When evaluating deaminase‐mediated BEs, non‐DNA off‐targets are an important factor to consider. Transcriptome‐wide RNA sequencing shows that early editors caused a lot of broad RNA deamination. Because of this issue, researchers developed SECURE variants to lower this risk [72, 259, 278, 280]. In the case of prime editing, methods like PE‐tag and AID‐seq were created to find pegRNA‐dependent off‐targets. These studies mostly show that the system has relatively high precision compared to Cas9 [294, 513]. For large‐scale integration that uses CASTs and serine integrases, Tn‐seq and ET‐seq are commonly used to map the insertion sites across the whole genome. It is quite important to use these approaches to tell apart the precise cut‐and‐paste insertions from the cointegrate products that still have vector backbones. The results indicate that Type I‐F CASTs show high specificity. However, Type V‐K systems can sometimes lead to a lot of off‐target integration [353, 356, 357, 363].

Finally, validation strategies have incorporated single‐cell multiomics and computational modeling to bridge the gap between genotype and phenotype. Perturb‐seq and CROP‐seq enable the high‐throughput validation of genetic perturbations by linking sgRNA identity to single‐cell transcriptomic outputs [283, 504]. Advanced iterations such as CRAFTseq and Direct Capture Perturb‐seq improve sensitivity by directly genotyping the target locus, enabling the distinction between monoallelic and biallelic edits and revealing subtle phenotypic shifts masked in bulk assays [263, 418, 514]. Functional validation of variants of uncertain significance (VUS) is achieved through saturation genome editing [515], which systematically introduces edits across essential genes to generate clinical‐grade evidence of pathogenicity [516, 517, 518]. These experimental workflows are increasingly standardized using computational tools such as CRISPResso for automated indel analysis and predictive models such as inDelphi [195, 519], which confirm that repair outcomes are deterministic based on local sequence context [175, 203, 520, 521, 522]. More recently, AI‐driven agents such as CRISPR‐GPT have demonstrated the capacity to autonomously design validation assays and interpret sequencing data [51, 52], effectively closing the loop between computational prediction and empirical verification [315, 523] (Figure 9C).

### Section summary

Enabling technologies play an important role in the link between theoretical genome writing and clinical translation. They help to change in vitro proofs of concept into clinical applications, which need to be physically deliverable, computationally optimized, and empirically verified. However, the field is still limited by persistent optimality trade‐offs. For example, AAVs have strict physical packaging limits and dose‐limiting immunogenicity. Besides, LNPs show quite limited efficacy for targeted extrahepatic delivery. At the same time, conventional short‐read sequencing is often unable to capture the large‐scale structural variants induced in vivo. It is expected that addressing these limitations will rely on the combination of advances in multimodal AI models, immunology, and materials science. Some emerging strategies involve the design of nonviral nanocarriers that are immune‐evasive and actively targeted, as well as the use of AI agents to autonomously predict and mitigate patient‐specific genomic instabilities. As computational autonomy and delivery mechanisms mature, engineering workflows may become more integrated, which could finally help to link in silico sequence generation and in vivo therapeutic implementation.

## 7 REFLECTIONS AND FUTURE DIRECTIONS

### Overview

Genome engineering is moving toward synthetic design and generative biology. This change is crucial because it moves from local gene modification to the editing of cellular genetic networks. To this end, researchers need to balance a few different factors. For example, we need to deal with the efficiency–precision–payload trilemma and reduce the immunogenic and genotoxic effects of large synthetic effectors. Besides, we have to design fully orthogonal biological systems through computational methods. We can now insert large exogenous bacterial complexes into human cells. However, if we use these active genome writers without enough optimization, it may lead to cellular toxicity and immune rejection. These risks show that the engineering methods need a lot of adjustments before widespread clinical use. In this section, we have reviewed the limits of the efficiency–precision–payload optimality trade‐off. We have also outlined the safety and immunogenicity challenges of genome writing and discussed the development of fully synthetic biological systems.

### The efficiency–precision–payload trilemma

Genome engineering changes from stochastic disruption to deterministic writing because we want to achieve precise control [524, 525, 526]. However, the field is still quite limited by a basic optimality trade‐off. This trade‐off means that it is very difficult to increase efficiency (frequency of edits in a population), precision (purity of outcomes and lack of off‐target effects), and payload (size and complexity of the intended modification) all at the same time. Most existing tools usually improve two of these parameters but limit the third one. Because of this, the biological limits of the application decide the choice of modality, and we lack a universal capability [527, 528].

For single‐nucleotide modification, this conflict is clear in the trade‐off between base editing (BE) and PE. BEs show the highest efficiency. They can reach an editing rate of over 90% in ex vivo systems, and this makes large‐scale functional screening [203] possible in diploid cells, because HDR is inefficient there [529, 530, 531]. However, this high efficiency will hurt precision and payload flexibility. The deaminase‐catalyzed mechanism often creates an activity window that leads to bystander edits. This makes genotype–phenotype interpretation more difficult because adjacent susceptible bases are altered [72, 521]. In addition, the kinetic optimization for better efficiency can lead to the random editing of transiently exposed DNA and RNA. For example, hyperactive variants like ABE8e have this problem. They need further engineering to achieve optimal specificity [107, 259, 262, 532]. Also, the payload scope of BE is quite limited to transition mutations. Therefore, a large part of nontransition substitutions and structural variants cannot be edited [513, 520, 533].

In contrast, PE solves some of the precision and payload problems of BE, but it is often limited by low efficiency. PE uses a template‐directed synthesis mechanism to avoid DSBs and bystander effects. It offers high‐fidelity correction and can introduce small insertions and deletions [35, 42, 534, 535, 536]. However, its efficiency is quite limited because the cellular MMR pathway usually cuts out the newly edited strand. PE is also easily affected by repressive chromatin environments [112, 537, 538, 539, 540]. Some strategies have improved the efficiency and increased the editing rates by almost 20 times [541]. For example, the coexpression of dominant‐negative MMR factors (MLH1dn) or the use of peptide binders (MLH1‐SB) play a role in this process. However, these methods introduce more complexity and potential toxicity [125, 287, 291, 542]. Therefore, we still face a choice between the high‐efficiency, low‐precision profile of deaminase‐based editors and the high‐precision, variable‐efficiency profile of reverse transcriptase‐mediated prime editors.

The optimality trade‐off is very clear in the field of genome writing, where the goal is to insert multikilobase payloads. Traditional DSB‐dependent approaches have a large theoretical payload capacity, but they hurt both precision and safety. This is because the repair process is stochastic and p53‐mediated toxicity is activated [6, 21, 62, 253]. To address the limitations detailed in Table 1, new non‐DSB technologies, like CASTs, separate writing from host repair pathways, and they reach almost complete integration efficiency in bacterial systems. However, when we try to use them in mammalian environments, we found a huge decrease in efficiency (<1%–5%). This shows a big gap between prokaryotic and eukaryotic systems [78, 371, 547, 548]. Besides, the flexible recruitment mechanisms of some CASTs can lead to intrinsic precision errors, or wobble, at the insertion site [337, 354]. Hybrid systems like PASTE and PASSIGE try to solve this problem by combining the precision of PE to define site‐specific attachment loci and the large payload capacity of serine integrases [39]. Although these methods work, the multistep processes are slow in kinetics. Therefore, they need the continual evolution of the recombinase (e.g., eeBxb1) to achieve therapeutic‐grade integration rates [39, 116, 273].

**Table 1:** Overview of strategies and mechanisms in precision genome engineering.

Technology category	Representative tools	Core mechanism	DNA repair dependency	Payload capacity	Key advantages	Primary Limitations & Genotoxicity Risks
Nuclease‐based Editing	SpCas9, Cas12a [5, 6, 7, 145, 146]	RNA‐guided DNA double‐strand breaks (DSBs) [61]	High (NHEJ, HDR, MMEJ) [13, 14, 15, 16, 62, 543]	Small indels (NHEJ); Variable inserts (HDR) [9, 16, 17, 19]	High cleavage efficiency; Broad targeting applicability [11, 12]	Structural genomic instability (large deletions, chromothripsis) [23, 24, 25, 26, 98, 99, 100]; p53 toxicity [20, 21, 22, 104]; Low HDR in post‐mitotic cells [94, 95]
Base Editing	CBEs (BE3/4), ABEs (ABE7.10/8e), GBEs [19, 33, 135, 259, 281]	Nickase + deaminase (Cytidine/Adenine) for targeted nucleotide transitions/transversions [72, 254]	Low (Mismatch repair and cellular glycosylases) [257, 258, 291]	Single nucleotide (1 bp) [255, 273, 274, 275]	DSB‐free; High efficiency in non‐dividing cells; High product purity (especially ABEs) [108, 109, 110, 111, 261]	Bystander edits [269, 270, 271, 272]; RNA/DNA off‐target deamination [243, 278, 279, 280]; Restricted primarily to specific substitution types
Prime Editing	PE2, PE3, PEmax, ePE [35, 77, 293, 294, 295]	Nickase + reverse transcriptase for template‐directed synthesis [289, 290]	Low (Flap excision/ligation, heavily antagonized by MMR) [287, 288, 291]	Small to medium (up to ~100 bp) [42, 534, 535]	DSB‐free; Can install all 12 base conversions, small indels without donor DNA [513]	Lower efficiency than BEs; Bulky editor size limits AAV delivery [467, 532]; Scaffold sequence incorporation and structural errors [510, 536, 542]
Integrase‐Mediated Writing	twinPE, PASTE, PASSIGE, eePASSIGE [39, 116, 273, 341]	Prime editing (att site installation) + Serine integrase (e.g., Bxb1)[376, 377]	None	Large (~ 1 to 36 kb) [39, 378]	Programmable multi‐kilobase insertion; Avoids endogenous HDR and cell‐cycle constraints [81, 378]	Slow kinetics; Requires large, multi‐component delivery; Persistent attL/attR scars [82, 116, 544]
RNA‐Guided Transposition	CASTs (Type I‐F VchINT, Type V‐K ShCAST) [37, 38, 78, 352, 545]	Nuclease‐deficient Cas + transposase machinery (TnsA/B/C, TniQ) [353, 354, 366]	None	Large (up to ~10‐15 kb) [356, 363]	High integration efficiency (in bacteria); Cut‐and‐paste (I‐F) enables precise insertions [37, 363]	Low efficiency in mammalian cells [369]; Cointegration of vector backbones (V‐K) [524, 525, 526, 527, 528]; Wobble at insertion sites [337, 354]
Programmable bRNA Systems	IS110 bRNA [40, 41, 82, 397]	Single recombinase guided by bispecific bridge RNA (target & donor loops) [40, 398, 546]	None	Large (~ 5 kb verified, potentially larger) [40, 399]	Hypercompact homodimer; Truly RNA‐programmable recombination (no specific att/PAM sequences); Independent of host factors [41, 103]	Currently primarily validated in bacterial and cell‐free systems; In vivo specificity needs further optimization [40, 148]
Epigenetic Editing	CRISPRoff/on, dCas9‐KRAB, dCas9‐p300/Tet1 [46, 47, 301, 314, 316]	dCas9 + Epigenetic modifiers (e.g., Dnmt3a/3 L, KRAB, LSD1) [119, 217]	None	Heritable state (No DNA sequence change) [47, 120, 192]	Reversible modulation via CRISPRon [73, 134, 319]; Heritable silencing memory; Completely avoids DNA damage	Potential memory erasure by host surveillance in early development [318]; Requires specific cellular contexts for long‐term stability

Abbreviations: AAV, adeno‐associated virus; ABEs, adenine base editors; att, attachment site; bp, base pair; bRNA, bridge RNA; CASTs, CRISPR‐associated transposases; CBEs, cytosine base editors; dCas9, catalytically inactive Cas9; DSBs, double‐strand breaks; eePASSIGE, evolved and engineered PASSIGE; ePE, engineered prime editor; GBEs, glycosylase base editors; HDR, homology‐directed repair; indels, insertions and deletions; kb, kilobase; KRAB, Krüppel‐associated box; MMEJ, microhomology‐mediated end joining; MMR, mismatch repair; NHEJ, nonhomologous end joining; PAM, protospacer adjacent motif; PASSIGE, prime editing‐assisted site‐specific integrase gene editing; PASTE, programmable addition via site‐specific targeting elements; PE, prime editing/prime editor; twinPE, twin prime editing.

Finally, another problem of this trade‐off comes from the physical payload of the editor itself. Advanced editing machineries, like PEmax or fusion‐based integrase systems, are often too large for the packaging limits of standard delivery vectors like AAV [112, 467, 532]. This causes another compromise between the effector size and activity. Hypercompact nucleases like Cas12f and IscB provide a solution to the delivery bottleneck, but they usually show much less efficiency than the larger nucleases [153, 348, 433]. Consequently, extensive rational engineering or split‐intein delivery strategies are required to restore activity, further increasing manufacturing complexity [36, 112, 155]. It remains a big challenge to solve this trade‐off for the next generation of genome engineering [130, 148] (Figure 10A). We expect that generative AI‐driven design of compact, high‐efficiency, and precise synthetic editors may be a way out, but the specific mechanisms are unknown [550].

**Figure 10:**
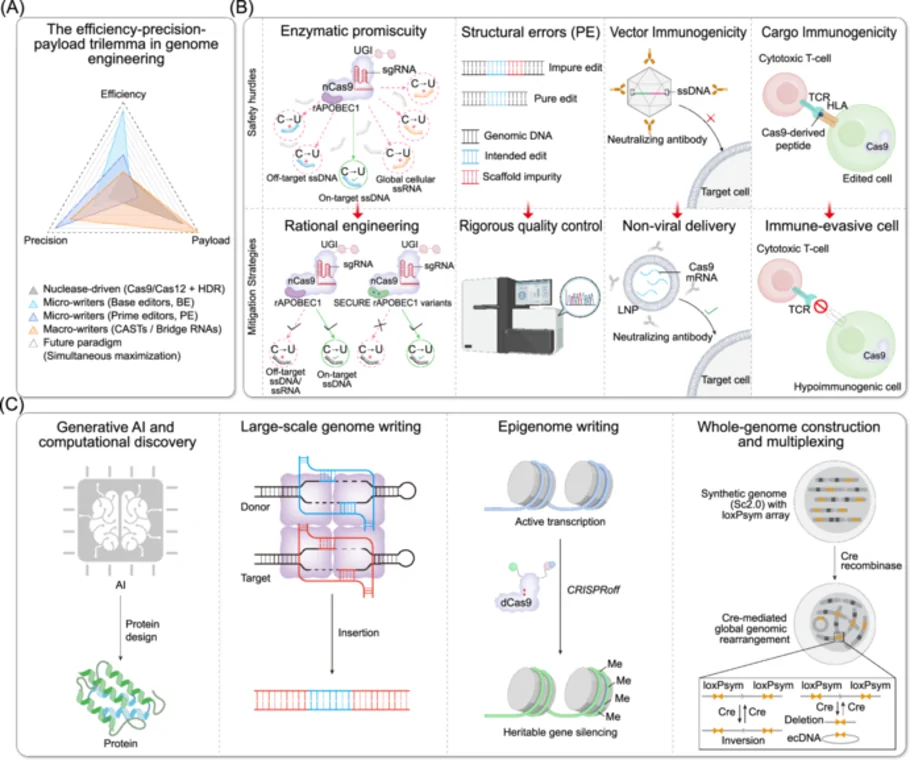
Reflections and future directions in precision genome engineering. (A) The efficiency–precision–payload trilemma. Current modalities, including nuclease‐driven editors, micro‐writers like base and prime editors, and macro‐writers such as CRISPR‐associated transposases (CASTs) and bridge RNAs, inherently compromise specific parameters to optimize others. Future paradigms require simultaneous maximization across all three dimensions. (B) Safety hurdles and corresponding mitigation strategies. Enzymatic promiscuity of deaminases induces off‐target DNA mutations and transcriptome‐wide RNA deamination [280], which are minimized through rational engineering of high‐fidelity variants. Structural errors during prime editing, including scaffold sequence incorporation [536], necessitate rigorous quality control. Furthermore, vector immunogenicity and cargo‐specific cytotoxic T‐cell responses are circumvented by utilizing transient nonviral delivery platforms, including lipid nanoparticles, and engineering hypoimmunogenic cells via human leukocyte antigen depletion [549]. (C) Paradigms of generative biology and synthetic design. Emerging frontiers encompass generative artificial intelligence (AI) for computational protein design [49] and programmable bridge RNAs that mediate site‐specific recombination between target and donor DNA. Additionally, CRISPRoff systems enable targeted epigenome writing for heritable transcriptional silencing, while whole‐genome multiplexing leverages synthetic chromosomes containing loxPsym arrays to drive Cre‐mediated global genomic rearrangements [396].

### Safety and immunogenicity in the era of writing

The shift from random gene disruption to exact genome writing indicates that current safety standards need to be re‐evaluated. Writing technologies like base editing, prime editing, and integrase‐mediated insertion were primarily developed to avoid the damage caused by DSBs. However, these tools introduce their own set of risks, which are related to structural, enzymatic, and immunogenic factors. For clinical applications, researchers must find ways to limit these side effects and manage the host immune response to the bacterial components required for carrying large genetic payloads.

A major safety issue with early nuclease tools is the genomic instability caused by DSBs [194]. For example, Cas9 cleavage can lead to severe genomic alterations. These changes are not just limited to small indels but also include megabase‐scale chromosomal truncations and chromothripsis, in which chromosomes shatter and randomly reassemble [23, 24, 25, 551]. In sensitive contexts, such as human embryos, DSB induction often leads to whole‐chromosome loss or loss of heterozygosity, rather than the intended repair [98, 205]. In addition, these breaks activate a strong p53‐mediated response, which can arrest the cell cycle or induce apoptosis in target cells. This also provides a survival advantage for p53‐deficient cells, raising concerns about tumorigenesis in patients [20, 22, 104]. Although nickase‐based tools are considered safer [552, 553], they still carry risks. For instance, DNA nicking can lead to replication fork collapse during the S phase and generate single‐ended DSBs. If the repair process is incorrect, this can result in large deletions or translocations [26, 554, 555]. Besides, prime editing also requires attention, because it may produce impure edits, such as scaffold sequence incorporation or template duplication. Thus, strict quality control is needed for any written sequence [100, 510, 536, 542].

Apart from structural problems, the enzymes involved in genome writing can show promiscuous activity and cause unintended damage. Early CBEs based on rat APOBEC1 lead to Cas9‐independent DNA mutations. They also induce RNA deamination across the transcriptome, which affects the expression of many genes [243, 279, 295]. Similarly, ABEs may self‐edit and show RNA activity, creating a mixture of modified proteins with unpredictable behaviors [32, 278, 324]. To reduce these risks, current efforts rely on rational engineering [139, 556]. Newer variants like SECURE editors and those containing the evolved TadA‐8e are much more effective at decoupling DNA editing from RNA affinity, bringing off‐target activity close to background levels [276, 278, 557, 558]. In the case of mitochondrial editing, the bacterial toxin DddA is used. However, it requires a split‐protein architecture to prevent cellular toxicity and limit off‐target damage [327, 330, 336]. Despite these improvements, validation is still difficult. Computational models frequently miss off‐target sites generated by individual genetic variations. Therefore, empirical methods, such as DISCOVER‐Seq, are necessary for safety evaluation [80, 245, 508].

The application of genome writing in patients is further limited by the immune system. Viral vectors, particularly AAVs, trigger immune reactions that prevent repeated dosing. They can also lead to insertional mutagenesis. AAVs frequently integrate at DNA damage sites, which may activate oncogenes [102, 103, 559]. Furthermore, because Cas9 and related transposases are derived from bacteria, pre‐existing immunity is common in the human population. This can result in the clearance of edited cells [99, 110, 484]. To circumvent these barriers, the focus has shifted to nonviral delivery methods, such as LNPs. Because LNPs are only present in the body for a short time, they help to reduce off‐target editing and immune responses, which makes readministration possible [485, 489, 560]. Other strategies are also being investigated. For example, in utero editing can induce immune tolerance to the treatment. Another approach is to generate hypoimmune cells by deleting human leukocyte antigen (HLA) genes, which helps to manage the host reaction against these foreign systems [549, 561, 562].

Finally, to ensure safety, it is critical to control the exact timing of editor activity. The discovery of anti‐CRISPR proteins (Acrs) provides a method to inhibit Cas9 and Cas12a. This approach helps to restrict off‐target damage and enables the reversal of epigenetic modifications [73, 134, 563]. Studies show that these proteins function by mimicking DNA to block nuclease activity [74, 446, 564, 565, 566]. However, the long‐term stability of these synthetic circuits remains uncertain. In bacterial populations, escaper mutants can overcome the CRISPR system, which shows the potential limitations of these tools [311, 499, 536, 567]. As a result, future therapeutic strategies will likely rely on transient delivery. The goal is for the machinery to perform its function and degrade rapidly. In this way, the genomic modifications are retained, while the writer itself is cleared from the cell, as shown in Figure 10B [205, 248, 568].

### The synthetic future

Genome‐engineering technologies have evolved from early gene surgery, which focused on correcting discrete errors in endogenous sequences, to a new stage called genome writing. This new approach is defined by the deterministic insertion of complex genetic information and the de novo design of biological functions [6, 524]. This change is an important step in biotechnology, as researchers move from reading and editing the code of life to actively writing it. Ultimately, genome writing moves beyond the mere modification of existing genetic text, unlocking the capacity to build genomes, or massive regulatory segments thereof, entirely from the ground up [28, 30]. We expect that this capability will lead to the engineering of complex multigenic traits and the restructuring of entire genomes [56]. Also, it plays a role in creating synthetic biological systems with capabilities beyond natural evolutionary limits.

However, achieving this synthetic future is quite limited by the efficiency–precision–payload optimality trade‐off. In many cases, tools perform well in one dimension but sacrifice the others. For example, early nucleases showed high efficiency but lacked precision. However, HDR provided precision, but its efficiency was much less in nondividing cells [6, 150]. Recently, new approaches have started to address these problems. Prime editing uses a template‐directed synthesis capability. It can precisely write small insertions and all base‐to‐base conversions without DSBs or donor DNA. This method may theoretically address approximately 89% of pathogenic variants [35, 513]. We can overcome the payload limitations of prime editing by combining it with recombinases. Systems like PASTE use an integrase‐mediated translocation mechanism to insert cargo as large as 36 kb, and they show almost no detectable off‐target integration. Later modifications to this approach, such as eePASSIGE, optimized serine integrases through continuous evolution. This system achieves very high integration efficiencies for 10‐kb payloads in primary human fibroblasts, which outperforms previous methods by 16‐fold [116, 273].

At the same time, we recently found bRNAs in IS110 elements. This finding introduces a fully programmable recombination mechanism, which avoids the complex protein–DNA recognition codes of traditional transposases. This system physically links target and donor DNA through bispecific RNA loops to perform modular insertion, excision, and inversion. Many consider this a third generation of RNA‐guided architecture [397]. Together, these technologies provide the mechanical capacity to write multigenic circuits and therapeutic modules. This is much more complex than merely correcting single nucleotides.

The ability to write large sequences has unlocked the engineering of polygenic traits and complex metabolic networks, moving beyond a monogenic targeting approach. Notably, industrial biotechnology researchers have achieved system‐level reprogramming of cellular metabolism. Multiplex knockout of *LDHA* and four PDK isozymes has created Warburg‐null mammalian cells that rely on oxidative phosphorylation rather than aerobic glycolysis, eliminating lactate production in order to stabilize biomanufacturing processes [91, 92]. Similarly, MAGE has been used to simultaneously modify 24 genetic components to optimize lycopene production in *E. coli* [59, 392], while CASTs have been deployed in vivo to write metabolic pathways into native gut bacteria that confer novel fiber‐utilization abilities controllable by diet [56, 371]. In agriculture, multiplex editing has produced poplar trees with a 51.7% reduction in lignin [569] and enabled the de novo domestication of orphan crops, such as groundcherry by rapidly engineering compact architecture and increasing fruit size [332, 570], and facilitated polyploid genome design in crops through the creation of clonal gametes to harness progressive heterosis [571].

Building and changing whole genomes is an important hot spot in this field. For example, the Sc2.0 project made a designer yeast chromosome V. It has hundreds of loxPsym sites to help inducible evolution, which shows that synthetic genomes can be chemically made to match in silico designs with single‐nucleotide precision [55, 395, 569]. In mammalian systems, methods like HACE lead to continuous, targeted hypermutation of long genomic intervals. This affects the directed evolution of drug resistance and splicing logic in endogenous contexts [409, 411]. Besides, scientists adapted the SCRaMbLE system to human cells using prime editing. This change produces stochastic megabase‐scale rearrangements, which suggests that the human genome has enough plasticity to survive large‐scale synthetic restructuring [396]. Similarly, targeted chromosome engineering has been extended to plants; by inducing CRISPR‐Cas‐mediated breaks at subcentromeric and subtelomeric regions, researchers successfully generated heritable chromosome fusions in *Arabidopsis thaliana*, reducing the chromosome number from 10 to 8 without affecting vegetative phenotypes or transcription profiles [572]. This ability also plays a role in making genomically recoded organisms with synthetic auxotrophy. It can work as a relatively stable biocontainment mechanism [57, 399, 573, 574].

Writing functional states and historical records is also an important part of the synthetic future. For example, tools like CRISPRoff help the programmable writing of heritable epigenetic memory. Because of this, genes can be silenced for hundreds of cell generations in permissive cellular contexts, and the underlying DNA sequence remains unchanged [47, 192]. Conversely, dCas9‐based systems influence multiplex gene activation. This can lead to cellular differentiation or changes in gene expression across a quite wide dynamic range [53, 305, 416]. With these changes, the genome is acting more like a dynamic recording medium. Systems like mSCRIBE and DNA Typewriter use self‐targeting guides or prime editing to write analog biological histories into DNA arrays. This method makes cells work like flight recorders, which show developmental and pathological processes [403, 405, 406, 407, 417].

In the medical field, the focus is shifting, from fixing specific mutations in single patients to new strategies that rely on universal gene replacement and functional engineering. Clinical trials show that it is possible to write personalized, neoantigen‐specific TCRs into patient T cells [242, 492]. However, ex vivo therapies have big economic problems. These issues lead to a shift toward in vivo delivery and whole‐gene replacement strategies. We hope these methods can treat almost all variants of a disease using just one therapeutic product [114, 235, 575]. Besides, high‐throughput variant‐to‐function screens are making a big difference in diagnostics. When researchers write thousands of variants into cells, they can classify VUS and predict drug responses before the patients show clinical symptoms. This change will considerably alter precision medicine [22, 35, 520, 576].

These technologies, when used in conjunction, display the foundation of generative biology. Because of the development of genomic foundation models like Evo and agentic automation systems like CRISPR‐GPT, the field of genome engineering is changing fast. It has moved to the computational codesign of proteins, regulatory elements, and even whole genomes [50, 51, 52]. The synthetic future may not be limited by nature. Instead, it relies on the ability to design and write biological systems that have novel chemistries, orthogonal dependencies, and better functions. We expect that these abilities can play an important role in dealing with problems in health, sustainability, and industrial production (Figure 10C).

### Section summary

The development of the genome‐writing era shows an important change in status. Biology is not just a system to study and fix anymore. It may become a programmable substrate, which can be computationally designed, assembled, and run *de novo*. However, this vision remains limited by the realities of host biology, where increasing synthetic cargo size is consistently associated with diminished in vivo efficiency. At the same time, persistent and highly immunogenic foreign enzymatic payloads can cause cellular instability, and this problem limits their therapeutic viability. To solve these biological limits, we may need to use closed‐loop robotic platforms and multi‐agent AI systems. These tools can help iteratively codesign sequences and delivery vehicles, producing ultracompact, hypoimmunogenic, and transiently active molecular machines, which make precise modifications and then degrade quickly. These changes lead to a new goal: a time of universal in vivo gene replacement and more dynamic biological operating systems. Under this condition, the genetic code of living organisms may be modulated safely and precisely. This can influence how we solve complex challenges in human health and biotechnology.

## 8 CONCLUSION

As shown in this review, the progress of genome engineering shows a fundamental change. We are moving from the random disruption of the genetic code to its controlled rewriting. In the early days, the field mainly relied on the idea of gene surgery. However, the unpredictable results of endogenous DNA repair and the toxicity of DSBs severely limited this approach. Now, researchers have developed various genome writers. These tools can change genes without relying on the physiological repair pathways of the host. Besides, scientists use the modular features of mobile genetic elements to achieve this. For example, prime editing depends on reverse transcriptase to ensure precision, whereas bRNAs and CASTs participate in programmable recombination. Because of these methods, we can slowly overcome the old trade‐offs between efficiency, precision, and payload capacity. Working together, these tools allow us to precisely insert multikilobase regulatory logic and metabolic pathways. Therefore, the genome is no longer just a static record. It plays a role as a functional medium that we can use for complex engineering.

In the future, combining generative AI with high‐throughput screening will likely lead to the rise of generative biology. This means we may use computational design to bypass the limits of natural evolutionary history. We are slowly shifting from finding natural enzymes to designing de novo editors that show non‐natural properties, such as hypercompactness and orthogonal specificities. This change may help us deal with the current delivery bottlenecks, and it can also widen the therapeutic window for universal gene‐replacement strategies. Today, genome engineering does much more than just correct single‐nucleotide errors. It can synthesize entire functional modules and also participate in recording biological histories. In fact, the field is turning into a way to process biological information. If this trend continues, we expect to have a much greater ability to program biological functions. The precision, scalability, and logical structure of this process will be quite similar to writing computer code.

## AUTHOR CONTRIBUTIONS

**Kerui Huang:** Conceptualization; writing—original draft; writing—review and editing; visualization. **Jianhong Tian:** Conceptualization; writing—original draft; writing—review and editing; visualization. **Wenyan Zhao:** Writing—original draft; writing—review and editing; visualization. **Siqin Zhang:** Writing—original draft; writing—review and editing; visualization. **Lixuan Xiang:** Writing—original draft; writing—review and editing; visualization. **Meiqing Yang:** Writing—original draft; writing—review and editing. **Lu Liu:** Writing—original draft; writing—review and editing. **Yazhou Huang:** Writing—original draft; writing—review and editing. **Ningyun Zhang:** Writing—original draft; writing—review and editing. **Lei Sun:** Writing—original draft; writing—review and editing. **Peng Xie:** Writing—original draft; writing—review and editing. **Nanyu Kuang:** Writing—original draft; writing—review and editing. **Hong Zhou:** Writing—original draft; writing—review and editing. **Chengfeng Qiu:** Writing—original draft; writing—review and editing. **Jun Yan:** Writing—original draft; writing—review and editing. **Xiaocheng Jiang:** Writing—original draft; writing—review and editing. **Qiang Tu:** Conceptualization; writing—original draft; writing—review and editing; visualization; supervision; funding acquisition. **Yun Wang:** Conceptualization; writing—original draft; writing—review and editing; visualization; supervision. **Haoliang Hu:** Conceptualization; writing—original draft; writing—review and editing; visualization; supervision. **Youming Zhang:** Conceptualization; writing—original draft; writing—review and editing; visualization; project administration; supervision; funding acquisition.

## CONFLICT OF INTEREST STATEMENT

The authors declare no conflicts of interest.

## ETHICS STATEMENT

No animals or humans were involved in this study.

## Data Availability

No new data were created or analyzed in this study. Supplementary materials (graphical abstract, slides, videos, Chinese translated version, and updated materials) may be found in the online DOI or iMeta Science http://www.imeta.science/imetaomics/.
